# Selected miRNAs in Urinary Extracellular Vesicles Show Promise for Early and Specific Diagnostics of Diabetic Kidney Disease

**DOI:** 10.1002/jex2.70089

**Published:** 2025-10-14

**Authors:** Karina Barreiro, Jenni Karttunen, Erkka Valo, Essi Viippola, Ileana Quintero, Annemari Käräjämäki, Antti Rannikko, Harry Holthöfer, Andrea Ganna, Niina Sandholm, Lena M. Thorn, Per‐Henrik Groop, Tiinamaija Tuomi, Om Prakash Dwivedi, Maija Puhka

**Affiliations:** ^1^ Institute for Molecular Medicine Finland FIMM, HiLIFE University of Helsinki Helsinki Finland; ^2^ Institute for Molecular Medicine Finland FIMM, HiPREP Core University of Helsinki Helsinki Finland; ^3^ Folkhälsan Research Center Helsinki Finland; ^4^ Department of Nephrology University of Helsinki and Helsinki University Hospital Helsinki Finland; ^5^ Research Program for Clinical and Molecular Metabolism, Faculty of Medicine University of Helsinki Helsinki Finland; ^6^ Department of Primary Health Care, Vaasa Central Hospital, Vaasa, Finland & Diabetes Center Vaasa Health Care Center Vaasa Finland; ^7^ Research Program in Systems Oncology, Faculty of Medicine University of Helsinki Helsinki Finland; ^8^ Department of Urology University of Helsinki Helsinki University Hospital Helsinki Finland; ^9^ Department of Obstetrics and Gynecology University of Helsinki and Helsinki University Hospital Helsinki Finland; ^10^ Department of General Practice and Primary Health Care University of Helsinki and Helsinki University Hospital Helsinki Finland; ^11^ Department of Diabetes Central Clinical School, Monash University Melbourne Victoria Australia; ^12^ Baker Heart and Diabetes Institute Melbourne Victoria Australia; ^13^ Lund University Diabetes Centre, Department of Clinical Sciences Lund University Malmö, Sverige Sweden; ^14^ Endocrinology, Abdominal Centre Helsinki University Hospital Helsinki Finland

**Keywords:** biomarkers, diabetes, diabetic kidney disease, liquid biopsies, small RNA sequencing, urinary extracellular vesicles

## Abstract

Diabetic kidney disease (DKD) is a health burden that lacks specific and early diagnostic biomarkers. For their discovery, we sequenced urinary extracellular vesicle miRNAs in a type 1 diabetes cohort of males with and without DKD. The results were replicated by sequencing or qPCR in two independent cohorts and six published datasets, including type 1 and 2 diabetes, and both sexes. We also validated stable reference gene candidate miRNAs. Chronic kidney disease, hypertensive nephropathy, IgA nephropathy, polycystic kidney disease, kidney stones, prostate cancer and non‐diabetic cohorts served as additional controls. MiRNAs changed due to urine collection type or centrifugation before storage were excluded. We analyzed differentially expressed miRNAs and their correlations with clinical measurements, receiver operating characteristic curves and target mRNAs, proteins and pathways, incorporating single‐cell data and circulating proteins of type 1 and 2 diabetes cohorts. By studying the uEV miRNAs (*N* = 490 individuals total) and plasma proteins (*N* = 4335), we pinpointed 6 stable miRNAs, 11 differentially expressed miRNAs, 9 target proteins and 16 DKD‐associated pathways. Differentially expressed miRNAs overlapped between diabetes subtypes and sexes, with strongest evidence for miR‐192‐5p, miR‐146a‐5p, miR‐486‐5p and miR‐574‐5p. The miRNAs alone or combined with clinical measurements classified individuals with the fastest kidney function decline (sensitivity 0.75–1.00, specificity 0.83–1.00) even in the normoalbuminuria group. The differentially expressed miRNAs did not cluster the control cohorts except for the chronic kidney disease cohort, which showed some clustering based on proteinuria status. Altogether, the miRNAs showed potential to identify early kidney function decline and may target key kidney cells, mRNAs, proteins and pathogenic mechanisms in DKD.

## 1 Introduction

Diabetes is a growing health burden that affects close to 10% of the world population (Saeedi et al. 2019). The major challenge of diabetes is that the devastating complications cannot be sufficiently prevented or cured. Diabetic kidney disease (DKD) is a common microvascular complication of ∼30% of the individuals with type 1 diabetes (T1D) (Jansson Sigfrids et al. 2022; Tuttle et al. 2022) and ∼40% of those with type 2 diabetes (T2D) (Tuttle et al. 2022). The factors leading to DKD include hyperglycemia, oxidative stress, chronic inflammation, obesity, insulin resistance and hypertension, but also genetic factors (Jung and Yoo 2022; Lyssenko and Vaag 2023; Sandholm et al. 2023). Of note, the DKD susceptibility and disease course differ between the sexes (Clotet‐Freixas et al. 2024; Loeffler and Ziller 2023) and diabetes subtypes (Ahlqvist et al. 2018). DKD manifests as progressive albuminuria and a relentless decline in the kidney function measured by estimated glomerular filtration rate (eGFR) (Thomas et al. 2015). As the diagnosis currently relies on the rather late and somewhat nonspecific biomarkers, albuminuria and eGFR, the kidneys are already to some extent damaged at the time of diagnosis (Thomas et al. 2015). Kidney biopsies could provide an accurate diagnosis, but kidney biopsies are rarely performed due to the cost and risk of complications (Gonzalez Suarez et al. 2013; Poggio et al. 2020). Novel, early and specific diagnostics would enable early interventions to protect the kidney—combating the progression of DKD to kidney failure.

Urinary extracellular vesicles (uEV) offer a potential non‐invasive diagnostic solution. The uEVs are secreted nanovesicles that carry a rich RNA cargo reflecting the pathophysiological state of the originating genitourinary cells and tissues (Erdbrügger et al. 2021). Sex affects the uEV secretion and cargo: males produce more kidney‐derived uEV and generally uEV with partly different nucleic acids than females (Ben‐Dov et al. 2016; Blijdorp et al. 2022; Dwivedi et al. 2023; Sedej et al. 2022). Our previous DKD studies indicated that the uEV represents a useful source of kidney miRNAs and mRNAs (Barreiro et al. 2023; Dwivedi et al. 2023). In both sexes, uEV carries RNAs showing correlations with, for example, eGFR, albuminuria, and chronic kidney disease (CKD) stage (Barreiro et al. 2023; Dwivedi et al. 2023; Zapała et al. 2023). However, several technical challenges and unanswered questions in uEV‐based DKD research remain. The uEV studies have been generally small and have applied variable preanalytical, analytical, and clinical study designs (Erdbrügger et al. 2021; López‐Guerrero et al. 2023). DKD‐linked uEV miRNAs have been studied more in T2D than in T1D. Furthermore, exploration of the candidate markers in multiple cohorts or control groups has been rare. Therefore, the biomarker candidates have varied, even if some results appear initially replicable (Barreiro et al. 2023). Thus, our aim was to understand whether the uEV could provide specific miRNA biomarkers primarily for early but also for later DKD and for different diabetes subtypes, sexes or particular pathogenic pathways. With uEV miRNA sequencing, we not only discovered miRNAs linked with DKD, declining kidney function and pathogenic mechanisms, but also validated stable reference candidate miRNAs across patient cohorts and preanalytical variables.

## 2 Materials and Methods

### 2.1 Clinical Data and Urine Samples From Diabetes Cohorts

Urine samples and clinical data for the discovery cohort and replication cohorts were collected in the nationwide prospective Finnish Diabetic Nephropathy (FinnDiane) Study and the Diabetes Registry in Vaasa (DIREVA study) as described (Dwivedi et al. 2023) (Table 1). Clinical data are presented for the T1D discovery and replication cohorts in Tables 2 and 3, respectively, and for the T2D replication cohort in Table 4.

**TABLE 1:** Cohorts profiled by miRNA sequencing in this study.

Cohort	Collection type	Storage Temp	PI *	Pre‐Clearing	uEV isolation Method	DNAse treatment	Disease and sex	*n* (donors)	Previous omics profiling
Discovery cohort (FinnDiane & DIREVA)	24 h/ON	−80°C	Yes	Yes‐No **	UC	No	Non‐diabetic and T1D. All males.	Non‐diabetic = 7 Normoalbuminuria = 40 Microalbuminuria = 15 Macroalbuminuria = 19	mRNA‐seq (Dwivedi et al. 2023)
Replication cohort T1D (FinnDiane)	24 h	−80°C	Yes	Yes	UC	Yes	T1D. All females.	Normoalbuminuria = 23 Microalbuminuria = 4 Macroalbuminuria = 4	mRNA‐seq (Dwivedi et al. 2023)
Replication cohort T2D (DIREVA)	ON	−80°C	Yes	Yes	UC	No	T2D. Females and males.	Microalbuminuria = 14 (2 females) Macroalbuminuria = 9 (1 female)	mRNA‐seq (Dwivedi et al. 2023)

*Note*: Overnight urine collections (ON), protease inhibitors (PI), type 1 diabetes (T1D), type 2 diabetes (T2D), ultracentrifugation (UC), urinary extracellular vesicles (uEV). ** 24 h urine collections were not pre‐cleared, but overnight urine collections were pre‐cleared.

**TABLE 2:** Clinical characteristics of the male T1D discovery cohort stratified into normo‐, micro‐ and macroalbuminuria groups that were used in global differential expression analysis.

		Normoalbuminuria		Microalbuminuria		Macroalbuminuria		Non‐diabetic
	*N*	38	*N*	15	*N*	19	*N*	7	*p* value
Age (years) (18–70)	37	50.63 (18.32–73.85)	15	49.36 (27.50–69.87)	19	49.97 (29.03–67.91)	7	52.26 (31.59–62.00)	0.979
BMI (kg/m^2^)	36	26.40 (20.25–38.58)	15	27.61 (21.50–40.03)	19	27.69 (19.88–38.49)	7	27.83 (24.01–30.97)	0.642
WHR	36	0.94 (0.78–1.08)	15	0.94 (0.85–1.09)	18	0.99 (0.87–1.13)	7	0.93 (0.84–1.03)	0.067
HbA1c (mmol/mol)	37	57.97 (36.00–73.00)	15	64.27 (45.00–87.00)	19	69.58 (47.00–85.00)	7	35.00 (32.00–39.00)	<0.001
SBP (mmHg)	37	136.80 (113.00–165.50)	15	141.33 (115.50–168.50)	19	149.05 (118.00–188.50)	7	131.57 (111.00–159.50)	0.096
DBP (mmHg)	37	76.07 (58.50–87.50)	15	80.23 (64.00–93.00)	19	79.45 (66.50–102.50)	7	77.71 (65.50–91.50)	0.340
eGFR (ml/min/1.73 m^2^)	35	96.30 (69.81–130.96)	15	96.42 (51.20–120.44)	19	67.30 (12.52–110.94)	7	86.72 (70.88–108.66)	0.004
Diabetes duration (years)	35	32.81 (6.57–57.81)	15	36.10 (18.92–49.36)	19	36.52 (19.00–48.39)			0.454
AER (mg/24 h) *	14	0.93 (0.08–4.00)	3	6.17 (0.61–18.10)	9	86.59 (17.59–197.80)			<0.001
AER (µg/min) #	16	6.85 (0.01–24.09)	8	57.27 (29.88–119.30)	2	254.28 (29.56–479.00)			<0.001
Hypertension (*N*)	14	7	5	5	13	12	7	2	
Retinopathy (*N*)	14	3	5	3	13	11	7	0	
Medication ACE inhibitor/AT2R blocker (*N*, users)	14	3	7	7	15	11	7	1	

*Note*: *p* Values were calculated using Kruskal‐Wallis statistics. Pairwise comparisons are shown in Table S1a. Angiotensin‐converting enzyme inhibitors (ACE inhibitors), angiotensin 2 receptor blockers (AT2R blocker), diastolic blood pressure (DBP), estimated glomerular filtration rate (eGFR), glycated haemoglobin (HbA1c), systolic blood pressure (SBP), waist‐to‐hip ratio (WHR), *albumin excretion rate (AER) (mg/24 h) as measured in FinnDiane; #AER (µg/min) as measured in DIREVA, *N* = total numbers.

**TABLE 3:** Clinical characteristics of the female T1D replication cohort stratified into normo‐, micro‐ macroalbuminuria and albuminuria (micro‐ and macroalbuminuria) groups.

		Normoalbuminuria		Microalbuminuria		Macroalbuminuria			Albuminuria	
	*N*	23	*N*	4	*N*	4	*p* value	*N*	8	*p* value
Age (years) (21–60)	23	46.11 (21.07–59.17)	4	44.74 (30.29–55.70)	4	47.82 (33.51–54.44)	0.706	8	46.28 (30.29–55.70)	0.547
BMI (kg/m^2^)	23	28.58 (19.38–41.12)	4	30.29 (23.33–37.38)	4	27.13 (24.86–28.51)	0.710	8	28.71 (23.33–37.38)	0.910
WHR	23	0.85 (0.74–1.09)	4	0.92 (0.80–1.02)	4	0.84 (0.79–0.91)	0.338	8	0.88 (0.79–1.02)	0.353
HbA1c (mmol/mol)	23	60.18 (44–75)	4	63.25 (51–76)	4	63.43 (61–66)	0.660	8	63.34 (51–76)	0.380
SBP (mmHg)	23	128.50 (99.00–162.50)	4	127.38 (105.00–154.00)	4	125.88 (117.00–134.50)	0.936	8	126.63 (105.00–154.00)	0.748
DBP (mmHg)	23	78.61 (63.50–94.00)	4	76.13 (69.50–81.50)	4	75 (69.50–88.00)	0.547	8	75.56 (69.50–88.00)	0.337
Diabetes duration (years)	23	32.02 (6.59–53.75)	4	29.72 (26.52–36.38)	4	37.39 (27.85–45.02)	0.488	8	33.55 (26.52–45.02)	0.804
eGFR (ml/min/1.73 m^2^)	23	99.36 (47.87–130.26)	4	62.57 (52.10–75.42)	4	58.01 (15.78–103.69)	0.004	8	60.29 (15.78–103.69)	<.001
AER (mg/24 h)	23	11.96 (4.25–50.06)	4	38.91 (16.80–67.89)	4	772.34 (32.40–1747.30)	<.001	8	405.62 (16.80–1747.30)	<.001
Hypertension (*N*)	23	12	4	3	4	4		8	7	
Retinopathy (*N*)	23	8	4	2	4	4		8	6	
Medication ACE inhibitor/AT2R blocker (*N*)	23	12	4	3	4	4		8	7	

*Note*: *p* Values were calculated using Kruskal–Wallis statistics or Mann–Whitney statistics. Pairwise comparisons are shown in Table S1b. Albumin excretion rate (AER), angiotensin‐converting enzyme inhibitors (ACE inhibitors), angiotensin 2 receptor blockers (AT2R blockers), diastolic blood pressure (DBP), estimated glomerular filtration rate (eGFR), glycated haemoglobin (HbA1c), systolic blood pressure (SBP), waist‐to‐hip ratio (WHR).

The studies followed the principles of the Declaration of Helsinki, and all participants gave informed consent prior to participation. The study protocol for this substudy of the FinnDiane and DIREVA studies was approved by the Ethics Committee of the Turku University Hospital. The study protocol of the FinnDiane study has also been approved by the Ethical Committee of the Helsinki and Uusimaa Hospital District (FinnDiane 491/E5/2006; 238/13/03/00/15; HUS/3313/2018), and that of DIREVA, by the Ethics Committees of the Vaasa Central Hospital (6/2007) and Turku University Hospital (48/1801/2014; 116/1805/2016).

### 2.2 Stratification of Participants Into Normo‐, Micro‐ and Macroalbuminuria Groups

In the FinnDiane study, stratification was done according to the AER (urine albumin excretion rate) (based on timed urine collections) in two out of three consecutive urine collections: macroalbuminuria with AER >300 mg/24 h or >200 µg/min, microalbuminuria with AER 30–300 mg/24 h or 20–200 µg/min, and normoalbuminuria with AER <30 mg/24 h or <20 µg/min. In the DIREVA study, macroalbuminuria was defined as AER (based on overnight urine collection) >200 µg/min or ACR (spot urine albumin/creatinine ratio) >35 mg/mmol, and microalbuminuria as AER 20–200 µg/min or ACR 3.5–35 mg/mmol at the last clinical visit, except for a few borderline cases based on longitudinal values. Normoalbuminuria was defined as AER <20 µg/min and ACR <3.5 (mg/mmol) at all the clinical visits.

### 2.3 Clinical Data Collection

Research nurses or physicians collected information on diabetes heredity, history of pancreatitis or gestational diabetes, and, by using standardized questionnaires, on sex, age, age at onset of diabetes, duration of diabetes, micro‐ and macrovascular complication profile and current medication. Measurements of height, weight, body‐mass index (BMI), waist and hip circumference, waist‐to‐hip ratio (WHR), blood pressure, glycated haemoglobin (HbA1c), blood lipids and lipoproteins (total and HDL‐cholesterol and triglycerides), creatinine, fS‐C‐peptide, fP‐Glucose, S‐GAD‐antibodies (only DIREVA) and DNA were collected in both studies as explained previously (Dwivedi et al. 2023). We also obtained retrograde data on HbA1c, serum and plasma creatinine for estimation of glomerular filtration rate (eGFR using the CKD‐EPI formula) (Levey et al. 2009), AER, and urine albumin‐creatinine ratio (ACR) from hospital laboratory databases for the validation of the albuminuria status as well as for the longitudinal analyses.

To calculate the eGFR slope, 8–15 years of retrospective data and the average of all eGFR values per year for each individual were used. The last measurement was obtained close to the time point of the urine collection. The eGFR slopes were estimated by linear regression analysis. The eGFR values used for the clinical measurement correlations and the ROC analysis were measured close to the urine collection time point.

### 2.4 Extracellular Vesicle Isolation and Analysis

For T1D discovery cohort and T2D replication cohort uEV were isolated as previously described (Dwivedi et al. 2023), 30 mL urine was centrifuged (15 min, 8000 × *g*, +4⁰C), filtered with 1.2 µm cellulose acetate (Whatman, GE Healthcare, Buckinghamshire, UK) or 1.2 µm PES filters (Pall Medical, Fribourg, Switzerland) and ultracentrifuged at 100,000 × *g* (27 500 rpm) for 1.5 h using SW32 rotor with Beckman L‐70 Optima ultracentrifuge and thickwall polypropylene tubes (Beckman Coulter, Inc., Brea, CA, USA). The pellet was washed, and the final pellet suspended in filtered PBS after recentrifugation before freezing at −80⁰C.

For the T1D replication cohort, EVs were collected from 7.8 mL of preprocessed urine (done as above) with ultracentrifugation at 100,000 × *g* (25,700 rpm, 1.5 h, +4⁰C) using a Beckman L‐70 Optima ultracentrifuge with 70.1 Ti rotor and thick‐wall polypropylene tubes (Beckman Coulter). After supernatant removal, the pellet was suspended in filtered PBS and frozen at −80°C.

The uEVs were analyzed using transmission electron microscopy (TEM) as described (Puhka et al. 2017) and with Immunoelectron microscopy (IEM), nanoparticle tracking analysis (NTA), and single particle interferometric reflectance imaging sensing (SP‐IRIS) as described below.

### 2.5 Immunoelectron Microscopy

IEM was performed for one representative sample of each group (non‐diabetic, T1D with normoalbuminuria, and T1D with microalbuminuria, presenting the albuminuric group). Tetraspanins were labelled with antibodies for CD9 (#AFC‐132rft‐0.1, clone MEM‐61, Nordic BioSite), CD81 (#AFC‐HZA1BE‐0.1, clone M38, Nordic BioSite) and CD63 (Pelicluster CD63, clone CLB‐gran/12, 435, Sanquin). The stainings were performed as previously described (Puhka, Takatalo, et al. 2017) with a small modification. Briefly, uEVs were loaded to grids, fixed with 2% paraformaldehyde, and blocked with 0.5% BSA and 0,1% saponin in 0.1 M phosphate buffer (pH 7.0). Primary and 10 nm gold‐conjugated secondary antibodies (BBI Solutions, Cardiff, UK) were diluted in 0.1% BSA, 0.05% saponin, 0.1 M phosphate buffer in a 1/50 and 1/80 ratio, respectively. After embedding, the uEV samples were assessed using a transmission electron microscope, Hitachi HT7800, operating at 100 kV. Images were acquired with a Gatan Rio9 bottom‐mounted CMOS camera (model 1809, Gatan Inc.) with 3072 × 3072 pixel image size and no binning.

### 2.6 Nanoparticle Tracking Analysis

Particle concentration and size were measured with the ZetaView PMX‐120 NTA instrument (Particle Metrix GmbH, Ammersee, Germany) equipped with a Z NTA cell assembly, a blue (488 nm, 40 mW) laser, and a CMOS camera with 640 × 480‐pixel resolution. Samples were diluted in a total volume of 1 mL of particle‐free ultra‐pure milli‐Q water to obtain 50–200 particles per frame. Videos in NTA mode were recorded at 11 positions across the measurement chamber in 2 s increments at 30 FPS framerate with camera shutter speed at 100/s and sensitivity at 85. The temperature was controlled at 22°C for NTA. Videos were processed and outliers (>10% CV) were removed using the Grubbs method with the built‐in ZetaView software (version 8.05.12 SP2). Particles between 10–1000 nm in diameter with a minimum trace length of 15 frames and a minimum brightness of 20 were included in the analysis.

### 2.7 Single‐particle Interferometric Reflectance Imaging Sensor

uEV samples were analysed with the Leprechaun Exosome Human Tetraspanin Kit with the ExoViewTM R100 instrument (NanoView Biosciences, Boston, MA, USA). The samples were diluted in incubation buffer to obtain a total particle count of 2 × 10^9^ based on NTA measurement. The chips were loaded with 35 µL of sample, and uEV were allowed to bind to antibody spots in sealed wells for 16 h at RT. The samples were stained with a mix of fluorophore‐conjugated antibodies against CD9, CD81 and CD63 labelled with CF‐488A, CF‐555 and CF‐647, respectively (provided in the kit). Chips were washed, dried, and scanned according to the manufacturer's instructions. The obtained data were analysed using the NanoViewer analysis software (NanoView Biosciences) version 3.0 with sizing thresholds set from 50 to 200 nm diameter on the interferometry channel. Fluorescence thresholds were set at 200 to 65536 A.U. on the red channel (CD63), 200 to 65536 A.U. on the green channel (CD81) and 480 to 65536 A.U. on the blue channel (CD9) by examining the level of background on spots coated with isotype controls.

### 2.8 RNA Isolation

For the discovery and T2D replication cohort sequencing, total RNA was isolated using miRNEasy mini kit (Qiagen, Hilden, Germany) without a DNAse treatment. The uEV samples of the T1D replication cohort for sequencing and all qPCR samples were treated with 5 U of DNAse I (Zymo Research, Irvine, CA) in PBS for 15 min at RT. Then, RNA was isolated using miRNEasy micro or mini kit (Qiagen). Concentrations and fragment lengths of all RNA samples were analyzed with RNA 6000 Pico Total RNA Kit run on Bioanalyzer 2100 (Agilent Technologies, Santa Clara, CA, USA).

### 2.9 Small RNA Sequencing

RNA sequencing libraries were generated using Lexogen Small RNA‐Seq library kit (Lexogen GmbH, Vienna, Austria) from 1 ng of RNA. Library quality was checked using Agilent Bioanalyzer High Sensitivity DNA assay (Agilent Technologies), and libraries were pooled based on the concentrations acquired from the assay. For discovery and T2D replication cohorts, no size selection was made. For the T1D replication cohort, adapter‐dimers were removed by size selection (130–180 bp) from pooled library using BluePippin (Sage Science, Beverly, MA, USA). The library pools were quantified using Collibri Library Quantification kit (Thermo Fisher Scientific, Waltham, MA, USA) and library fragment size was determined with LabChip GX touch system using High Sensitivity assay (Perkin Elmer, Waltham, MA, USA). Sequencing was performed on an Illumina NovaSeq6000 system using SP flow cell with lane divider (Illumina, San Diego, CA, USA). Read length for the single‐end run was 101 bp.

### 2.10 Small RNA Sequencing Data Analysis

Reads were trimmed using Trim Galore (version: 0.6.6) and aligned to the genome using COMPSRA (Comprehensive Platform for Small RNA Analysis) (Li et al. 2020) pipeline. STAR v2.5.3a (Dobin et al. 2013) was used as the sequence aligner by COMPSRA. Reads were first mapped to human hg38 genome using local type of read ends alignment and only one mismatch allowed. Aligned reads were then quantified and annotated by the COMPSRA annotation module using miRbase (https://mirbase.org/) (Kozomara et al. 2019) as reference database for miRNAs. All the downstream analyses of the obtained read counts were carried out using R, v. 4.0.3 (R Core Team 2020).

Principal component analysis (PCA) was performed using plotPCA function of DESeq2 and ggplot2 package (Wickham 2016). Based on the miRNA explorative plots, in the discovery cohort two and in the T1D replication cohort one normoalbuminuria samples were outliers and excluded from the downstream analysis.

For data visualization and analysis, the datasets were transformed using variance stabilizing transformation (VST), apart from correlations for clinical data, where normalized read counts were used. R version 4.2.1 (R Core Team 2022) and following packages were used: PCAtools package for PCA plots (Blighe and Lun 2025), EnhancedVolcano (Blighe et al. 2025) for volcano plots, pheatmap (Kolde 2025) for heatmaps, ggplot2 (Wickham 2016) for violin plots and ggpubr (Kassambara 2023b) and ggcorrplot (Kassambara 2023a) packages for correlation plots .

**TABLE 4:** Clinical characteristics of the T2D replication cohort stratified into micro‐ and macroalbuminuria groups.

		Microalbuminuria		Macroalbuminuria	
	*N*	14	*N*	9	*p* value
Age (years)	14	68.86 (61.00–78.00)	8	71.13 (56.00–81.00)	0.165
BMI (kg/m^2^)	14	30.91 (23.31–36.67)	8	33.46 (26.20–40.65)	0.212
WHR	14	1.07 (0.97–1.23)	8	1.07 (0.95–1.18)	0.920
HbA1c	14	60.86 (39.00–95.00)	8	67.38 (53.00–96.00)	0.267
SBP	14	138.00 (113.00–172.00)	7	151.57 (114.00–202.00)	0.287
DBP	14	81.86 (60.00–93.00)	7	78.86 (62.00–98.)	0.360
Diabetes duration (years)	14	19.5 (4.00–33.00)	8	18.63 (9.00–28.00)	0.714
eGFR (ml/min/1.73 m^2^)	14	63.11 (32.00–98.00)	8	62.63 (30.00–95.00)	0.868
ACR (mg/mmol)	14	18.14 (4.60–66.20)	8	96.03 (18.10–289.20)	0.005
Hypertension (*N*)	14	14	8	8	
Retinopathy (*N*)	13	3	7	2	
Medication ACE inhibitor/AT2R blocker (N, users)	14	14	8	8	

*Note*: *p* Values were calculated using Mann–Whitney statistics. Albumin‐to‐creatinine ratio (ACR)* measured from last laboratory visit, angiotensin‐converting enzyme inhibitors (ACE inhibitors), angiotensin 2 receptor blockers (AT2R blockers), diastolic blood pressure (DBP), estimated glomerular filtration rate (eGFR), glycated haemoglobin (HbA1c), systolic blood pressure (SBP), waist‐to‐hip ratio (WHR).

### 2.11 Quantitative PCR

For qPCR validation, uEV and RNA isolation were repeated for a set of original discovery cohort samples using a second urine aliquot. For the T1D replication cohort, original remaining RNA was used. Either 2 µL of original RNA or 1:2 to 1:5 dilutions (final amount 218–836 pg) were converted to cDNA and preamplified using TaqMan Advanced miRNA Synthesis Kit (Thermo Fisher Scientific) and T100 PCR machine (Bio‐Rad, Hercules, CA). A 3 µL of 1:10 (miR‐196b‐5p, miR‐574‐5p and miR‐143‐3p), 1:100 (miR‐222‐3p, miR‐24‐3p, miR‐192‐5p and let‐7c‐5p) or 1:1000 (miR‐23b‐3p, let‐7a‐5p, miR‐200c‐3p and miR‐204‐5p) dilution of the cDNA was mixed with 5 µL of TaqMan Fast Advanced Master Mix, 0.5 µL of miRNA specific TaqMan Advanced miRNA Assay (Table 5) and 1.5 µL of nuclease‐free water (all Thermo Fisher Scientific). Triplicate qPCR reactions were run on CFX96 real‐time PCR detection system, and the results were analyzed with CFX Maestro program (both BIO‐RAD).

**TABLE 5:** Details on TaqMan assays (Thermo Fisher Scientific) used for qPCR validation and normalization.

miRNA name	Assay ID	reference sequence
miR‐143‐3p	477912_mir	UGAGAUGAAGCACUGUAGCUC
miR‐222‐3p	477982_mir	AGCUACAUCUGGCUACUGGGU
miR‐574‐5p	479357_mir	UGAGUGUGUGUGUGUGAGUGUGU
miR‐196b‐5p	478585_mir	UAGGUAGUUUCCUGUUGUUGGG
miR‐192‐5p	478262_mir	CUGACCUAUGAAUUGACAGCC
miR‐23b‐3p	483150_mir	AUCACAUUGCCAGGGAUUACCAC
miR‐24‐3p	477992_mir	UGGCUCAGUUCAGCAGGAACAG
let‐7a‐5p	478575_mir	UGAGGUAGUAGGUUGUAUAGUU
let‐7c‐5p	478577_mir	UGAGGUAGUAGGUUGUAUGGUU
miR‐200c‐3p	478351_mir	UAAUACUGCCGGGUAAUGAUGGA
miR‐204‐5p	478491_mir	UUCCCUUUGUCAUCCUAUGCCU

To determine the qPCR assay efficiencies, we used uEV‐RNA from a non‐diabetic male and a male with T1D and macroalbuminuria. The pre‐amplified samples were serially diluted (from 1:10 to 1:1 × 10^6^) and qPCR efficiencies were calculated from ct values using the formula: E = −1+10(−1/slope) × 100. The slope was estimated using linear regression.

### 2.12 Replication Using Published miRNA Profiling Datasets

Sequencing and clinical data were obtained from the following publications or kindly provided by the publication authors upon request (see acknowledgements). Raw count data from Barreiro et al. 2020 were available from supplementary materials (Barreiro et al. 2020). Quantitative PCR data from Barutta et al. 2013 were available from NCBI Gene Expression Omnibus (GEO) repository with accession number: GSE48318. Raw count data from Ghai et al. 2018 was provided to us by the authors. Raw sequencing data from Park et al. 2020 and Park et al. 2022 were available from the NCBI BioProject repository, accession PRJNA736796 and accession PRJNA555060 (sample accession numbers: SRR14783956, SRR14783949, SRR14783947, SRR14783945, SRR9698095, SRR9698092, SRR9698086, SRR14783953, SRR14783951). Reads were preprocessed and aligned to the genome using excerpt (Rozowsky et al. 2019), specifically, exceRpt small RNA‐seq analysis pipeline (v 4.6.2) from exRNA tools in genboree.org (Coarfa et al. 2014; Subramanian et al. 2015). Analysis was run with default settings except for the following settings that were selected based on the recommendations given in the library preparation kit user manual: adapter sequence ‐AAAAAAAAAA, minReadLength ‐15 and trimBases5p ‐3. Raw sequencing data from Perez‐Hernandez et al. 2021 and Perez‐Hernandez, Riffo‐Campos, et al. 2021 were available in the NCBI BioProject repository, accession PRJNA590749. Reads were preprocessed and aligned using Chipster (Kallio et al. 2011). Alignment was done using bowtie (Langmead et al. 2009) default settings in Chipster except for ‐l 15, ‐k 1, and n‐mode. Raw sequencing data from Khurana et al. 2017 was available from ArrayExpress accession number E‐MTAB‐5152, and it was analyzed in Chipster in a similar manner than Perez–Hernandez data. Nanostring ncounter normalized data from Shankar et al. 2023 was available from GEO GSE244625. Raw sequencing data from Magayr et al. 2020 was available from arrayExpress accession number E‐MTAB‐8109 and was analyzed in chipster similarly to Perez‐Hernandez data. DESeq2 normalized counts, *p* values and fold changes (for the 11 differentially expressed miRNAs of our T1D discovery cohort) from Ali et al. 2024a and Ali et al. 2024b were provided to us by the authors. From this data, we selected the miRNAs with > 60% of normalized counts with value > 0 across all the samples (i.e., miR‐146a‐5p, miR‐192‐5p, miR‐93‐5p, miR‐23b‐3p, miR‐24‐3p). Raw counts from Zhu et al. 2024 were available from GEO accession number GSE241241. Normalized data of the prostate cancer data set from Puhka et al. 2022 was available in supplementary materials. Raw counts normalization and differential expression analysis from Barreiro et al. 2020; Ghai et al. 2018, Park et al. 2020, Park et al. 2022; Perez‐Hernandez et al. 2021; Khurana et al. 2017; Magayr et al. 2020; Zhu et al. 2024 data was done using DEseq2 (Love et al. 2014). Detailed information on the number of samples is presented in Table 6.

### 2.13 Linear Regression Models

Linear regression models to study the association of miRNAs with eGFR decline were created using the lm() function in R. Data were transformed to standard units using the scale function. Outliers, identified using qqnorm and boxplots, were removed. The models were corrected for age, diastolic blood pressure (DBP), HbA1C and systolic blood pressure (SBP).

### 2.14 Receiver Operating Characteristic Analysis

ROC analysis and plots were done using IBM SPSS Statistics V29 with nonparametric distribution assumption and confidence level of 95%. The ROC analysis of combinations of miRNAs validated in the T1D replication cohort (miR‐192‐5p, miR‐146a‐5p, miR‐486‐5p, and miR‐574‐5p) and clinical variables, that is, SBP, HbA1c and inverted estimated glomerular filtration rate (1/eGFR) was performed using the R package CombiROC (Mazzara et al. 2017). The models obtained with CombiROC using the discovery cohort data were used to classify the replication cohorts using the same R package.

Sensitivity and specificity reported for the confusion matrices were calculated using the following formula: sensitivity = true positives/(true positives + false negatives) and specificity = true negatives/(true negatives + false positives) as described (Hoyer and Zapf 2021).

### 2.15 Pathway Analysis

Differentially expressed (DE) miRNAs’ (from discovery cohort) mRNA targets were found using Ingenuity pathway analysis MicroRNA Target Filter (IPA, Qiagen) (accession date: 23.05.22). Only target genes with experimentally observed evidence and kidney expression were considered for further analysis. Diseases or functions associated with the target mRNAs were searched using IPA BioProfiler (accession date: 06.03.24). Here, only terms with human and mouse evidence were included, and to reduce the complexity of the analysis (number of nodes in the Sankey diagram), targets that were only associated with kidney cancer were excluded. Human kidney biopsy single‐cell sequencing data (Wilson et al. 2022) were used to assess whether the mRNA targets had been found to be DE in individuals with DKD. All data layers were visually integrated in a Sankey diagram built with Plotly in R (Sievert 2020).

### 2.16 Circulating Protein Targeted Analysis

We used plasma protein data from UK Biobank (Sun et al. 2023) to analyze the association between 35 proteins and renal and ophthalmic complications in individuals with T1D or T2D.

The proteomics data processing and quality control within the UK Biobank have been previously described (Sun et al. 2023). Briefly, the dataset included plasma proteomics samples from approximately 53,000 individuals and covered 2923 unique proteins profiled with the Olink Explore 3072 platform. We excluded individuals with missing genetic sex information and imputed missing values using the MissForest algorithm (Stekhoven and Bühlmann 2012), considering all available proteins in the imputation step.

We extracted the relevant ICD‐10 codes from inpatient visit data over the entire lifetime of the study participants. We considered the following ICD‐codes: Type 1 diabetes mellitus without complications (E10.9), Type 1 diabetes mellitus with renal complications (E10.2), Type 1 diabetes mellitus with ophthalmic complications (E10.3), Type 2 diabetes mellitus without complications (E11.9), Type 2 diabetes mellitus with renal complications (E11.2) and Type 2 diabetes mellitus with ophthalmic complications (E11.3).

For each protein, we used logistic regression to explore the association between protein levels and the two types of complications—renal and ophthalmic—conducting separate analyses for T1D and T2D. In each subanalysis, individuals with the complication of interest were designated as cases, while controls were required to have the respective type of diabetes and no specific complication. We conducted both sex‐stratified and sex‐combined analyses to explore the potential sex‐differential effect, with the exception of E10.2, where the sample size was not sufficient for sex‐stratified analyses. The models were additionally adjusted for the age at recruitment. All analyses were carried out in Python version 3.10.14.

### 2.17 Statistics

To estimate a sufficient sample size for the differential expression analysis of the discovery cohort, we used the R package ssizeRNA v.1.3.2 (Bi and Liu 2016). Setting the parameters to 1000 input genes, 20 DE miRNAs, fold change = 5, and FDR = 0.05, the sample size = 20 gave a power of 0.8.

For sequencing data, exploratory and differential expression analyses were conducted using the R package DESeq2 v. 1.32.0 (Stekhoven and Bühlmann 2012). Read count matrices were pre‐filtered to exclude small RNAs with <10 reads in total across all samples. For heatmaps, principal component analysis (PCA), and violin plots, data were transformed using variance stabilizing transformation (VST). MiRNAs that had a normalized mean count >5, adjusted *p* value (Benjamini‐Hochberg adjusted *p* value (Benjamini and Hochberg 1995) for multiple testing correction) <0.05, and absolute log2 fold change (FC) value >0.6 were considered differentially expressed (DE).

SP‐IRIS and qPCR data were analyzed using IBM SPSS Statistics V29 (IBM Corporation, Armonk, NY, USA). For SP‐IRIS, the percentage (%) data was arcsin transformed. To test tetraspanin colocalization differences between normoalbuminuria and albuminuria, a *t*‐test was used, and *p* values <0.05 were considered statistically significant. The qPCR data normality was tested with Shapiro–Wilk test. Statistical difference between normo‐, micro‐ and macroalbuminuria groups was calculated using one‐way ANOVA and Tukey HSD for post‐hoc multiple comparisons. Mean differences between normoalbuminuria and albuminuria (microalbuminuria + macroalbuminuria) groups were tested using an independent samples t‐test. MiRNAs were considered significantly DE with *p* values <0.05.

For all, in the Spearman correlation analysis, we considered correlations statistically significant if *R* >0.3 and *p* value <0.05. For linear regression model, beta coefficients were considered statistically significant if *p* values were <0.05.

For evaluation of the miRNAs that were not significantly DE in the published miRNA datasets, a higher or lower expression in the case versus control group was considered if the log2FC in sequencing was ≥0.6 or ≤−0.6, or the FC in qPCR was ≥2 or ≤−0.5, respectively. For circulating protein analysis, the *p* values were corrected for multiple testing using the Bonferroni correction.

## 3 Results

**FIGURE 1:**
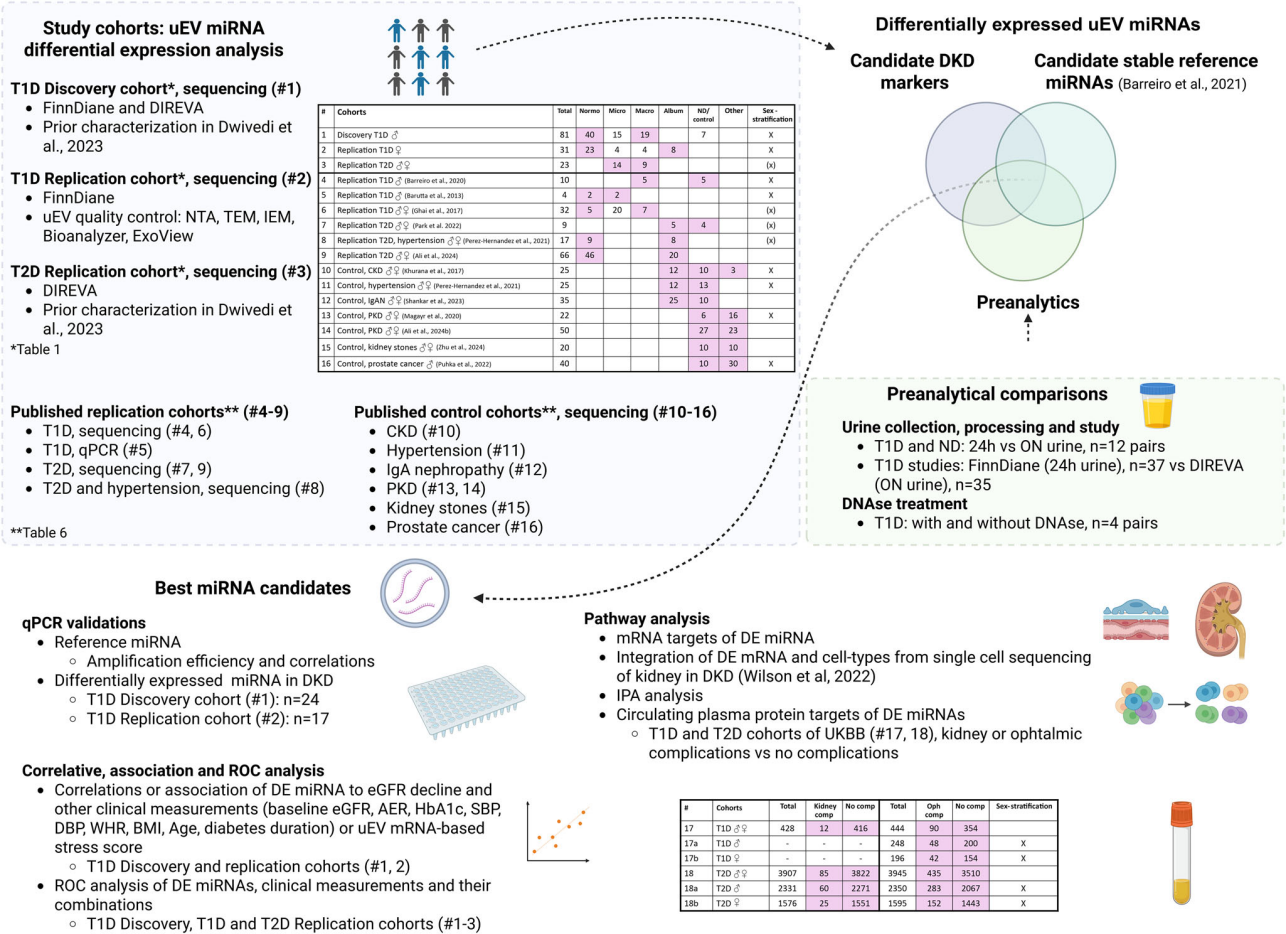
Study outline. The primary goal of the study was to discover urinary extracellular vesicle (uEV) miRNA markers for diabetic kidney disease (DKD) in individuals with T1D, with exploration in T2D. Differentially expressed (DE) miRNAs were analyzed in the discovery and replication cohorts (#1‐3) comprising different albuminuria groups and different timed urine collections, and between the preanalytical variables in the study. These data were cross‐checked for stable expression of our previously identified reference miRNA candidates (Barreiro et al., 2021). Candidate DKD marker and stable reference miRNAs were validated by qPCR. Markers were further explored in previously published uEV replication (#4‐9) and control (#10‐16) cohorts and through integrative pathway, circulating protein (cohorts #17, 18) and ROC analysis. Analysis of the different cohorts was carried out with sex‐stratification, where X=sex‐stratified differential expression or hierarchical clustering analysis, (x)=expressions plotted with sex‐colour coding, in addition to the analysis of the combined sex cohorts, whenever possible. Expression levels were correlated with clinical measurements altered in DKD, eGFR decline (slope) reflecting long‐term kidney function, and with a uEV‐mRNA based 'stress score', which we have found to be elevated in DKD (Dwivedi et al., 2023). Albuminuria (Album), albumin excretion rate (AER), body‐mass‐index (BMI), chronic kidney disease (CKD), complication (comp), diastolic blood pressure (DBP), estimated glomerular filtration rate (eGFR), haemoglobin A1c (HbA1c), immunoelectron microscopy (IEM), immunoglobulin A nephropathy (IgAN), macroalbuminuria (Macro), microalbuminuria (Micro), nanoparticle tracking analysis (NTA), non‐diabetic (ND), normoalbuminuria (Normo), ophthalmic (Oph), overnight (ON), polycystic kidney disease (PKD), receiver operating characteristic (ROC), systolic blood pressure (SBP), transmission electron microscopy (TEM), type 1 diabetes (T1D), type 2 diabetes (T2D), United Kingdom Biobank (UKBB), waist‐hip ratio (WHR). Partly created with BioRender.

We sequenced uEV miRNAs to identify miRNA biomarkers for DKD, focusing on T1D and reference miRNAs not significantly affected by common preanalytical variables (Figure 1). We applied differential expression analysis (1) for individuals with T1D with and without DKD, (2) for urine collection type, diabetes study and preanalytical processing and (3) cross‐checking these data for stable expression of our previously identified reference miRNA candidates (Barreiro et al. 2021). In the DKD study, we sequenced the T1D cohorts stratified by sex due to the potential sex‐based differences in DKD progression and uEV transcriptomes that is, we sequenced a T1D male discovery cohort and a T1D female replication cohort with matched clinical characteristics to provide insight into the miRNA changes in DKD by sex. The candidate reference and DKD marker miRNAs were confirmed by qPCR using both T1D cohorts. A mixed sex T2D cohort was sequenced to initially explore replication in T2D. DE miRNA expressions were then further analyzed in six previously published datasets, including two male and one mixed sex T1D cohorts and three mixed sex T2D cohorts. Chronic kidney disease, hypertensive and IgA nephropathy, polycystic kidney disease, kidney stones and prostate cancer cohorts served as additional controls. The controls were selected to study the specificity of the candidate miRNAs for DKD comprehensively, covering other proteinuric kidney diseases (CKD, hypertension and IgA), and a non‐glomerular CKD (PKD), as well as prostate cancer that represents another type of pathological genitourinary system condition that can be diagnosed based on the uEV transcriptome (Margolis et al. 2022; Wang et al. 2020).

The function of the candidate miRNA markers was explored through target mRNA and pathway analysis, incorporating kidney single‐cell transcriptomics data and circulating target proteins in T1D and T2D cohorts with and without sex‐stratification. The disease biomarker relevance was then explored by correlations and associations of the miRNA expression levels with eGFR decline reflecting the long‐term kidney function, clinical measurements altered in DKD, and a uEV‐mRNA‐based ‘stress score’, which we have found to be elevated in DKD (Dwivedi et al. 2023). The stress score was based on the transcriptional expression level of GPX3, NOX4, MSRB, MSRA, HRSP12 and CRYAB, as measured by mRNA sequencing. These genes were found to be upregulated in individuals with DKD and had functions associated with cellular and oxidative stress. In Dwivedi et al. 2023 we showed that the stress score reflected the long‐term kidney function decline. Utility of the miRNAs alone or combined with clinical measurements for identifying individuals with fastest declining eGFR was assessed with ROC plots.

### 3.1 uEV Preparation and Sequencing Quality

**FIGURE 2:**
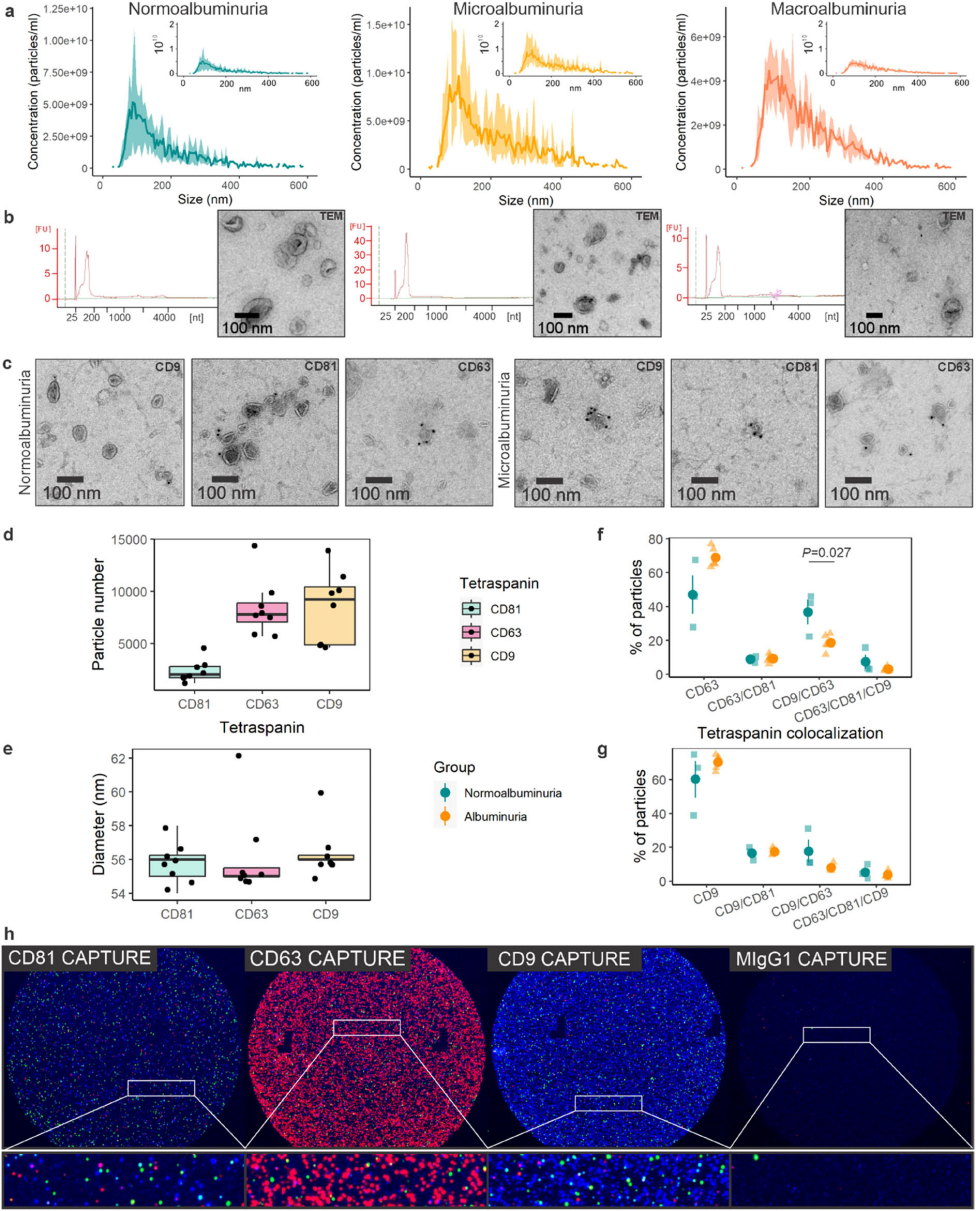
UEV characterization. (a) The particle size and concentration were measured using Particle Metrix Zetaview PMX‐120 nanoparticle tracking analysis (*n* = 3 per group). Histogram insets present the histograms with equal axis scales. (b) Representative Agilent total RNA pico chip electropherograms used to assess uEV RNA quality and quantity, and transmission electron microscopy (TEM) micrographs close‐ups revealing particles with cup‐shaped morphology in normo‐, micro‐, and macroalbuminuric samples, as in a. (c) TEM micrograph close‐ups showing the immunodetection of CD9, CD81 and CD63. Wide‐field micrographs for b and c are shown in Figure S1. (d–h) Characterization of uEV with ExoView platform (*n* = 8; normoalbuminuria *n* = 3, albuminuria *n* = 5). (d and e) Number and size of particles for CD81, CD63 and CD9 captured uEV. (f and g) Co‐localization analysis for CD63 and CD9 captured uEV, respectively. The round point and vertical line represent the mean ± standard error of the mean. Due to the low number of CD81 particles, colocalization analysis is only shown for CD63 and CD9 captures. (h) Representative images of CD81, CD63, CD9 and IgG isotype control spots on the ExoView chips. Coloured dots represent fluorescent detection of CD81 (green), CD63 (red) and CD9 (Blue) of particles captured in the spots. Urinary extracellular vesicles (uEV).

The uEVs were isolated and characterized as described (Dwivedi et al. 2023), except for the T1D replication cohort, where we used a protocol adopted for small urine volumes (Barreiro et al. 2021), and uEV quality was evaluated here. NTA showed 2.6 × 10^8^–3.1 × 10^9^ particles/mL of urine and a size distribution with a mean peak of 100 nm for all groups (Figure 2a). RNA presented typical uEV profiles: a main peak below ∼200 nt and occasionally, small ribosomal RNA peaks (Figure 2b). TEM revealed heterogeneous uEV <500 nm in diameter (Figure 2b) and only a few Tamm–Horsfall protein filaments. IEM and ExoView showed uEV positive for EV markers CD9, CD81 and CD63 (Figure 2c and Figure S1). Exoview showed less CD81 than CD9 or CD63 positive particles, but all had equal sizes (Figure 2d,e). Only CD63 and CD9 co‐positive particles were significantly decreased in the albuminuria samples (Figure 2f,g). Thus, the uEV preparation quality appeared fine.

### 3.2 Small RNA Sequencing Quality for Discovery and Replication Cohorts

In the discovery cohort, small RNA sequencing was performed for a total of 93 samples derived from the FinnDiane and DIREVA cohorts. Participants were all males and included 74 with T1D (2 outliers were excluded from subsequent analysis) and 7 non‐diabetic participants (Table 1). In 12 cases, both 24 h and overnight samples from the same patient and day were analyzed as preanalytical variants. Sequencing produced a median‐(IQR) of 3.6 (2.5–5.9) M raw sequencing reads, out of which median‐(IQR) 1.0 (0.6–2.3) M were aligned to miRNAs.

We previously found that the uEV‐RNA samples from females tend to have lower quality defined by characteristic RNA fragment length pattern generating less mapped transcriptomic reads and more intergenic reads compared to male samples (Dwivedi et al. 2023). Thus, for this study, we treated the uEV derived from the female T1D replication cohort with DNAse I. Given this protocol modification, we first assessed the effect of DNAse I using paired samples from females with and without the DNAse treatment (4 pairs covering different RNA qualities according to Dwivedi et al. 2023 and *n* = 2 normoalbuminuria, *n* = 2 albuminuria). We did not find statistically significant differences for the number of raw sequencing reads nor for the mapped reads (Figure S2a).

In replication cohorts, small RNA sequencing was performed for 31 samples of female participants with T1D from the FinnDiane Study and for 23 samples of male and female participants with T2D (Table 1). Here, for the female T1D cohort, sequencing produced a median‐(IQR) of 8.6 (6.2–13.1) M raw sequencing reads, out of which median‐(IQR) 1.9 (1.1–3.5) M were aligned to miRNAs. For the T2D cohort, sequencing produced a median‐(IQR) of 3.9 (2.0–6.8) M raw sequencing reads, out of which median‐(IQR) 0.9 (0.2–1.9) M were aligned to miRNAs.

The number of miRNAs detected, defined as miRNAs with average normalized expression of 5 across samples, was 291 for the discovery cohort, 423 for the T1D replication cohort and 261 for the T2D replication cohort.

Thus, a good amount of raw sequencing reads and detected miRNAs were obtained for all cohorts.

### 3.3 Comparisons Between miRNA Profiles From Different Long‐Term Diabetes Studies

**FIGURE 3:**
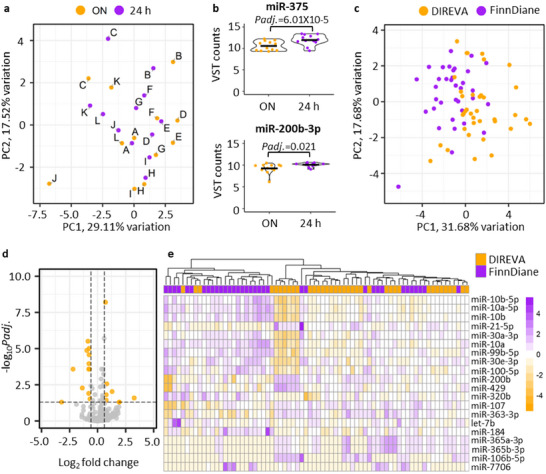
Preanalytical comparisons of the urine collections. First comparison included analysis of 12 paired samples with ON and 24 h collection time from the same individuals and day (4 non‐diabetic individuals and 8 individuals with T1D and DKD). (a) PCA analysis of the top 50 highest expressed miRNAs using VST normalized counts (raw counts were normalized and VST transformed using DeSeq2). Paired overnight and 24 h samples are marked with letters. (b) Differential expression analysis of the paired data resulted in two DE miRNAs. The expression values are represented in violin plots, and the black horizontal lines show the mean value. Second comparison included all T1D samples from FinnDiane versus DIREVA studies (FinnDiane *n* = 37 and DIREVA *n* = 35). (c) PCA analysis of the top 50 highest expressed miRNAs. (d) Volcano plot of miRNAs with mean expression level >5. (e) Heatmap clustering of the FinnDiane versus DIREVA samples based on the DE miRNAs. Overnight (ON), principal component analysis (PCA), Type 1 diabetes (T1D), variance stabilizing transformation (VST).

The FinnDiane and DIREVA cohorts used in this study differed regarding the original urine sample collection time and preprocessing of the samples before freezing. To evaluate the effect of these preanalytical variables on the uEV miRNA profiles we first compared overnight and 24 h collections (without centrifugation) from individuals with T1D and DKD manifesting as micro‐ or macroalbuminuria (*n* = 8) and non‐diabetic controls (*n* = 4). PCA performed using the top 50 highest expressed miRNAs did not separate the samples in clear groups (Figure 3a). The paired overnight and 24 h samples (marked in letters) were relatively close to each other but not perfectly overlapping. Differential expression analysis (Table S2a) revealed two DE miRNAs, miR‐375 and miR‐200b‐3p, when overnight was compared to 24 h collection time in a pair‐wise manner (Figure 3b).

Next, the FinnDiane and DIREVA cohorts were compared by using all the (unpaired) T1D samples. Here, overnight samples from the DIREVA study (*n* = 35) were centrifuged before freezing, whereas 24 h samples from the FinnDiane study (*n* = 37) were frozen without centrifugation. PCA from the top 50 highest expressed miRNAs showed some tendency of clustering, but no clear separation of the studies (Figure 3c). A total of 20 miRNAs were DE (Figure 3d, Table 2b). Heatmap clustering of these 20 DE miRNAs showed again some tendency but not clear separation of the studies (Figure 3e). The differential expression analysis included a correction for DKD status. We further controlled that the results did not arise from the DKD status by repeating the analysis for only the normoalbuminuria groups from the two studies (Finndiane *n* = 14 and DIREVA *n* = 24) (Table S2b). As a result, most of the DE miRNAs remained DE. Based on these results, we excluded these 22 preanalytically DE miRNAs when carrying out the DKD marker miRNA and reference miRNA studies.

### 3.4 Changes of the uEV miRNA Profile in DKD

**FIGURE 4:**
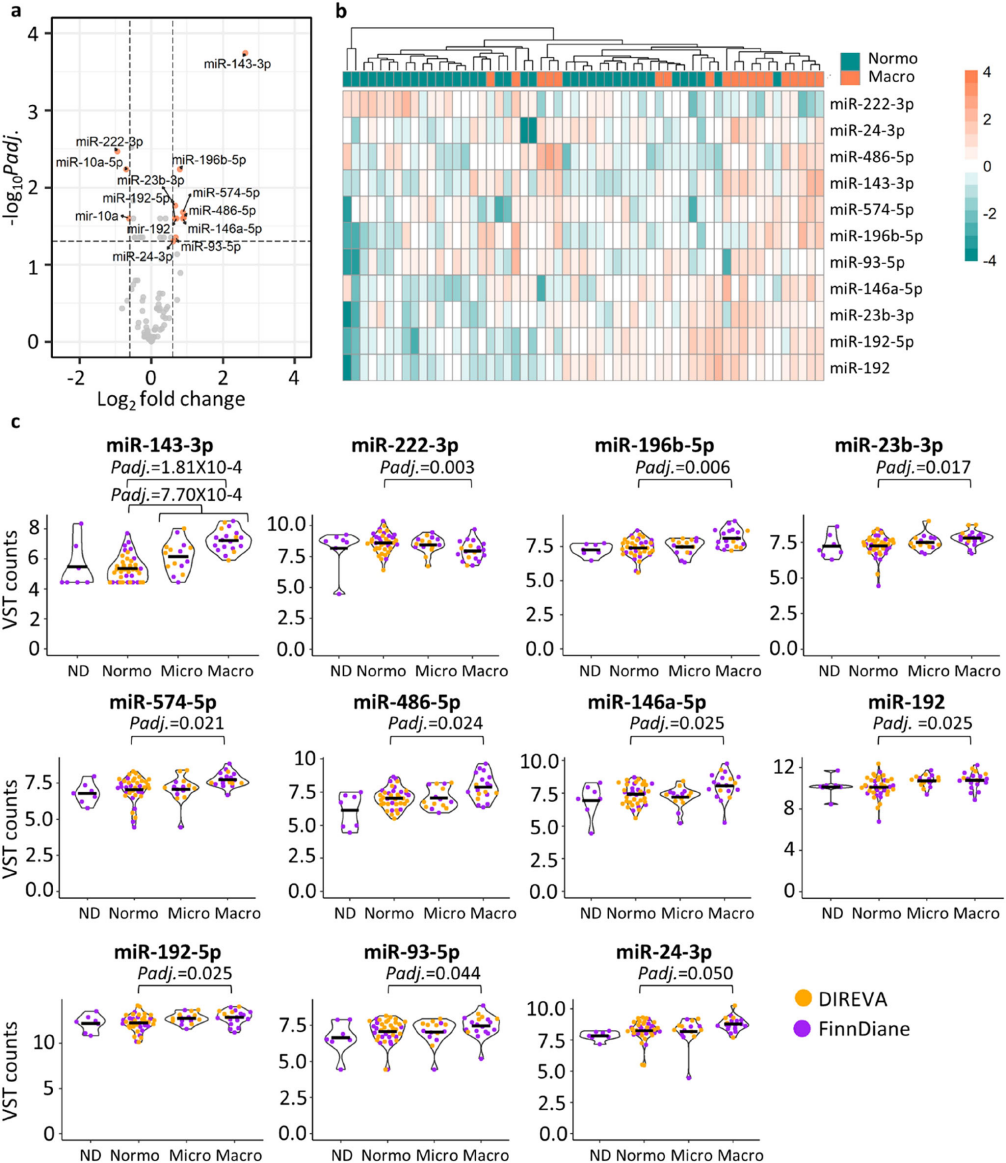
Differential expression analysis of miRNA sequencing data for the T1D discovery cohort. Differential expression between normoalbuminuric (*n* = 38) and macroalbuminuric groups (*n* = 19) (a and b). (a) Volcano plot presenting DE miRNAs (mean of five normalized counts, adjusted *p* value <0.05, and absolute log2FC >0.6). MiR‐10a and miR‐10a‐5p were DE in the preanalytical comparisons and were therefore not considered for further analysis. (b) Heatmap clustering based on the VST normalized read counts of the 11 DE miRNAs. (c) Violin plots from VST normalized data (raw counts were normalized and VST transformed using DeSeq2), including also non‐diabetic (*n* = 7) and microalbuminuria (*n* = 15) samples. The black horizontal lines show the mean value. Differentially expressed (DE), macroalbuminuria (Macro), microalbuminuria (Micro), non‐diabetic control (ND), normoalbuminuria (Normo), adjusted P‐value (Padj.), variance stabilizing transformation (VST).

Differential expression analysis was conducted with the T1D discovery cohort where clinical and demographic covariates (except for AER and eGFR) were matched between the study groups (non‐diabetic *n* = 7, normoalbuminuria *n* = 38, microalbuminuria = 15, macroalbuminuria = 19; Tables 1 and 2) and urine collection type was adjusted for in the DE analysis. The main analysis of macro‐ versus normoalbuminuria group—where sample size was reaching to the power of 0.8 according to the power calculation (methods)—revealed 11 significant DE miRNAs passing all criteria and when excluding changed miRNAs from the urine collection analysis: miR‐23b‐3p, miR‐24‐3p, miR‐93‐5p, miR‐143‐3p, miR‐146a‐5p, miR‐192, miR‐192‐5p, miR‐196b‐5p, miR‐222‐3p, miR‐486‐5p, and miR‐574‐5p (Figure 4a, Table S3b). Hierarchical clustering with the DE miRNAs showed clusters, although not a total separation of the groups (Figure 4b). The normalized counts of the DE miRNAs tended to increase from normo‐ to micro‐ and from micro‐ to macroalbuminuria (Figure 4c).

Differential expression analysis between the rest of the groups (Table S3c–e) showed significant upregulation of miR‐143‐3p in albuminuria versus normoalbuminuria comparison (Figure 4c). The differential expression analysis of macroalbuminuria versus non‐diabetic group (an additional control group) showed 9 DE miRNAs, including miR‐486‐5p and miR‐196b‐5p (Table S3f). The 11 DE miRNAs were further checked for any effect in expression when including or omitting the DNAse treatment. The comparisons showed that only miRNA‐486‐5p was higher expressed in the non‐DNAse treated samples (Figure S2b).

### 3.5 Correlation Analysis

**FIGURE 5:**
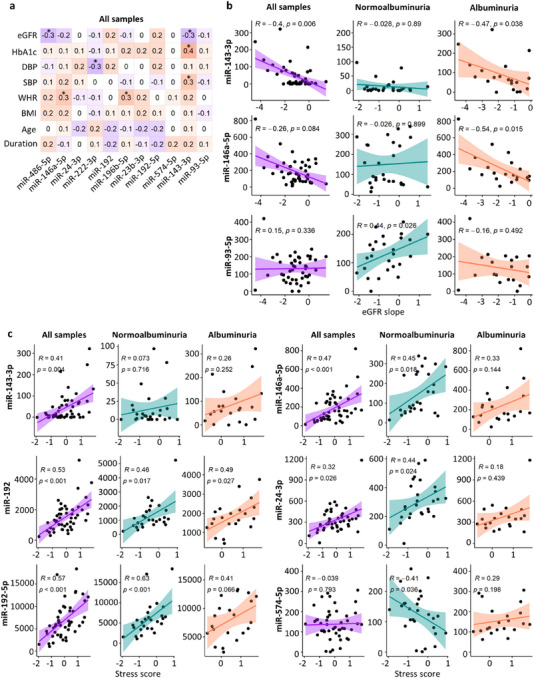
Correlation of normalized miRNA counts with clinical measurements and the stress score in the T1D discovery cohort (complete clinical data were available for 69 individuals). Correlations were considered statistically significant if R >0.3 and *p* <0.05. (a) Spearman correlation of each differentially expressed miRNA and clinical data collected at the time of urine collection. eGFR refers to baseline eGFR. p‐value < 0.05 (*). (b) Spearman correlation of eGFR slope data (eGFR decline speed) and those miRNAs that included at least one significant *p* value. eGFR slope data were available for 46 individuals with T1D (*n* = 26 normo‐, 10 micro‐, and 10 macroalbuminuria). (c) Spearman correlation of the stress score data and those miRNAs that included at least one significant *p* value. Albuminuria group refers to micro‐ and macroalbuminuria groups combined. The stress score data were available for 48 individuals with T1D (*n* = 27 normo‐, 10 micro‐, and 11 macroalbuminuria). Body mass index (BMI), diastolic blood pressure (DBP), estimated glomerular filtration rate (eGFR), glycated haemoglobin (HbA1c), macroalbuminuria (Macro), microalbuminuria (Micro), non‐diabetic control (ND), systolic blood pressure (SBP), waist‐hip ratio (WHR).

We next sought further proof of the relevance of the 11 DE miRNAs in DKD by correlating their expression with clinical measurements typically changed in diabetes and/or DKD. Analyzing all discovery cohort groups combined, significant but moderate correlations were found for miR‐143‐3p, miR‐146a‐5p, miR‐486‐5p, miR‐196b‐5p and miR‐222‐3p with eGFR, DPB, SBP, HbA1c or WHR (Figures 5a and S3).

To inspect the correlation with the eGFR slope, we analyzed all groups combined, the normoalbuminuria group alone, and the albuminuria group alone. MiR‐143‐3p correlated significantly with the eGFR slope in all groups combined and in the albuminuria group, miR‐146a‐5p in the albuminuria group and miR‐93‐5p in the normoalbuminuria group (Figure 5b).

We additionally analyzed, in the same groups, the correlation with the uEV “stress score”, an average expression value of six cellular and oxidative stress associated mRNAs, which was elevated in the individuals showing declining kidney function (Dwivedi et al. 2023). A significant correlation was found for miR‐192 in all three comparisons, and miR‐192‐5p, miR‐146a‐5p and miR‐24‐3p in all groups combined and in the normoalbuminuria group (Figure 5c). MiR‐143‐3p correlated significantly in all groups combined and miR‐574‐5p in the normoalbuminuria group (Figure 5c). Together, the DE miRNAs correlated with DKD‐associated parameters in different albuminuria groups alone or combined.

### 3.6 Replication Studies

**FIGURE 6:**
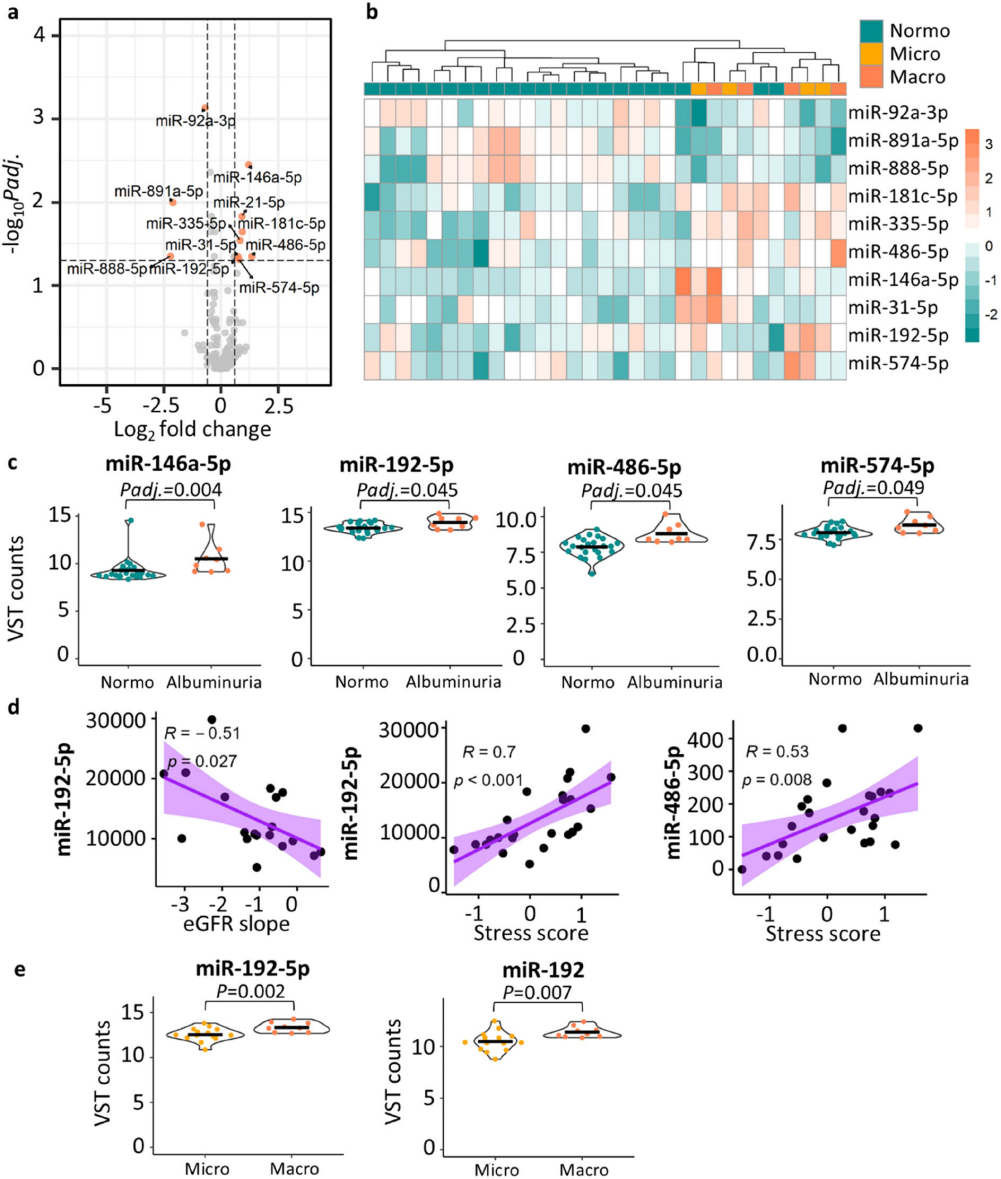
Replication of the miRNA biomarker candidates with an independent T1D female cohort and a T2D cohort. Sequencing results in the T1D female cohort (a–d) are shown as (a) volcano plot of all differentially expressed miRNAs (mean of five normalized counts, adjusted *p* value <0.05, and absolute log2FC >0.6) between albuminuria (*n* = 8) versus normoalbuminuria (*n* = 23) groups and (b) Heatmap clustering based on the VST normalized read counts of the 10 differentially expressed miRNAs not changed in the urine collection comparisons. (c) The expression levels (VST counts, raw counts were normalized and VST transformed using DeSeq2) in violin plots with a black horizontal line marking the mean value for the four differentially expressed miRNAs shared between the T1D discovery and replication cohorts. (d) Spearman correlation between the eGFR slope and miR‐192‐5p. The eGFR slope was available for 19 individuals (*n* = 16 normo‐, 2 micro‐, and 1 macroalbuminuria) and (d) Spearman correlation between the stress score and miR‐192‐5p or miR‐486‐5p. The stress score data were available for 24 individuals (*n* = 19 normo‐, 3 micro‐, and 2 macroalbuminuria). (e) Violin plots of VST counts for the two miRNAs with p‐nominal value <0.05 and absolute value of logFC > 0.6) shared between the T1D discovery and the T2D replication cohorts. Macroalbuminuria (Macro), microalbuminuria (Micro), normoalbuminuria (Normo), P‐adjusted value (Padj.), type 1 diabetes (T1D), variance stabilizing transformation (VST).

In the T1D replication cohort—where clinical and demographic covariates (except for AER and eGFR) matched between the study groups (Tables 1 and 3)—differential expression was analyzed between the albuminuria (*n* = 8, 4 micro‐ and 4 macroalbuminuria) versus normoalbuminuria (n = 23) groups combined to achieve better statistical power. Totally, 11 miRNAs were significantly DE out of which 10 were not changed in the urine collection comparisons (criteria as before, Figure 6a, Table S4). The 10 DE miRNAs showed a good separation of the normoalbuminuria and albuminuria groups (Figure 6b). Of note, using the 11 DE miRNAs from the male discovery analysis did not cluster the samples (Figure S4). MiR‐192‐5p, miR‐146a‐5p, miR‐486‐5p and miR‐574‐5p were similarly DE as in the discovery cohort (Figure 6c), whereas the rest—miR‐92a‐3p, miR‐891a‐5p, miR‐181c‐5p, miR‐335‐5p, miR‐888‐5p, miR‐31‐5p—were changed only in this female cohort. Additionally, miR‐192‐5p normalized counts correlated significantly with both the eGFR slope and the stress score (Figure 6c, d), and miR‐486‐5p correlated significantly with the stress score (Figure 6d).

Differential expression analysis using the T2D replication cohort that was again matched for clinical and demographic covariates (except for AER and eGFR) (Table 1 and 4) revealed an upregulation of the miR‐192 and miR‐192‐5p (nominal *p* value <0.05) between the macro‐ versus microalbuminuria groups (*n* = 14 and *n* = 9, respectively) (Figure 6e, Table S5, Figure S5).

To further validate the 11 DE miRNAs, we analyzed six published DKD studies with uEV miRNA sequencing or qPCR datasets. There, 10 miRNAs presented the same direction of change as in our data (Table 6, Figure S5).

**TABLE 6:** Published datasets (DKD, CKD or control) used to compare our findings.

Reference	miRNA profiling method	Diabetes type or control type, sex	Donors (*n*) (*n* females)	Higher expression in case group	Lower expression in case group	Figure
Barreiro et al. 2020	miRNA sequencing (QIAseq miRNA Library kit, Qiagen)	T1D, all male	ND = 5; macro = 5	**miR‐23b‐3p, miR‐143‐3p, miR‐146a‐5p, miR‐192‐5p, miR‐196b‐5p miR‐486‐5p**		Figure S5a
Barutta et al. 2013	qPCR (Human TaqMan miRNA Array A, Thermo Fisher Scientific)	T1D, all male	Normo = 2, micro = 2	**miR‐23b, miR‐143**		Figure S5b
Ghai et al. 2017	miRNA sequencing (in‐house pipeline)	T1D, combined	Normo = 5 (2 females); Overt nephropathy = 7 (2 females); Intermittent microalbuminuria = 9 (5 women); Persistent microalbuminuria = 11 (6 women)	miR‐23b‐3p*	miR‐146a‐5p, miR‐192‐5p, miR‐196b‐5p	Figure S5c
Park et al. 2020 and Park et al. 2022	miRNA sequencing (SMARTer smRNA‐Seq kit, Illumina)	T2D, combined	ND = 4 (0 females) T2D and DKD = 5 (2 females)	miR‐23b‐3p, miR‐146a‐5p*, miR‐192‐5p*, miR‐196b‐5p, miR‐574‐5p	miR‐486‐5p, miR‐93‐5p	Figure S5d
Perez‐Hernandez et al. 2021	miRNA sequencing (CleanTag Small RNA library preparation kit, TriLink Biotechnologies)	T2D, combined	T2D and hypertension without albuminuria (non‐UAE) = 9 (2 females); with albuminuria (UAE) = 8 (4 females)	miR‐93‐5p, miR‐143‐3p*, miR‐146a‐5p, miR‐486‐5p*	miR‐192‐5p*, miR‐196b‐5p, miR‐222‐3p*	Figure S5e
Ali et al. 2024	miRNA sequencing (QIAseq miRNA Library kit, Qiagen)	T2D, combined	T2D = 46; T2D and DKD = 20	miR‐23b‐3p, miR‐24‐3p, miR‐93‐5p*, miR‐146a‐5p*, miR‐192‐5p*		Figure S5f
Khurana et al. 2017	ncRNA sequencing (Ion Total RNA‐Seq Kit v2, Ion Torrent, Life Technologies)	Control, CKD, combined	Healthy control = 10 (5 females); CKD = 15 (6 females)	miR‐24‐3p*, miR‐143‐3p*, miR‐222‐3p*, miR‐486‐5p, miR‐574‐5p*		Figure 7a, S6a, and S6b
Perez‐Hernandez et al., 2021	miRNA sequencing (CleanTag Small RNA library preparation kit, TriLink Biotechnologies)	Control, hypertension, combined	Hypertension without albuminuria (non‐UAE) = 13 (5 females); with albuminuria (UAE) = 12 (4 females)	**miR‐23b‐3p, miR‐24‐3p, miR‐146a‐5p**	miR‐143‐3p, **miR‐222‐3p, ** miR‐574‐5p	Figure 7b and S6c
Shankar et al. 2023	Molecular barcoding and detection (NanoString nCounter Human v3 miRNA Expression Assay, NanoString Technologies)	Control, IgA nephropathy, combined	Healthy control = 10 (pooled samples, 1/4 females); IgA nephropathy = 25 (pooled samples, 1/4 females)	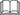 (none in top 10)	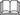 (none in top 10)	Figure 7c
Magayr et al. 2020	miRNA sequencing (Illumina TruSeq Small RNA Sample Preparation, Illumina)	Control, autosomal dominant polycystic kidney disease, combined	Healthy control = 6 (3 females); autosomal dominant polycystic kidney disease = 16 (7 females)		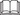 miR‐192*, miR‐93*	Figure 7d and S6d
Ali et al. 2024	miRNA sequencing (QIAseq miRNA Library kit, Qiagen)	Control, autosomal dominant polycystic kidney disease, combined	Healthy control = 28 (13 females); Autosomal Dominant Polycystic Kidney Disease = 23 (10 females)	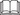 miR‐146a‐5p*		Figure 7e
Zhu et al. 2024	miRNA sequencing (NEBNext Multiplex Small RNA Library Prep Set for Illumina, NEB)	Control, kidney stones, combined	Healthy control = 10 (3 females); Kidney stones = 10 (3 females)	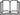 **miR‐23b‐3p, miR‐93‐5p,** miR‐222‐3p, miR‐574‐5p*	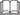 miR‐486‐5p	Figure 7f
Puhka et al. 2022	miRNA sequencing (QIAseq miRNA Library kit, Qiagen)	Control, prostate cancer, all male	Healthy control = 10; Prostate cancer (3 status and progression groups) = 30**	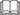 miR‐143‐3p*, miR‐146a‐5p*, miR‐192‐5p*, miR‐222‐3p*, miR‐486‐5p*, miR‐574‐5p*	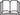 miR‐486‐5p*	Figure 7g

*Note*: Higher and lower expression (criteria in methods) of the 11 differentially expressed miRNAs from our DKD discovery study are shown for case groups in each study as analyzed by us or publication authors, or reported in the respective publication (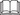). MiRNAs with direction of change in accordance with our findings are highlighted in bold. Macroalbuminuria (macro), microalbuminuria (micro), non‐diabetic (ND). *statistically significant regulation. **All changes reported, study contained many comparisons between different groups. From Barreiro et al. 2020 we used the ultracentrifugation dataset.

**FIGURE 7:**
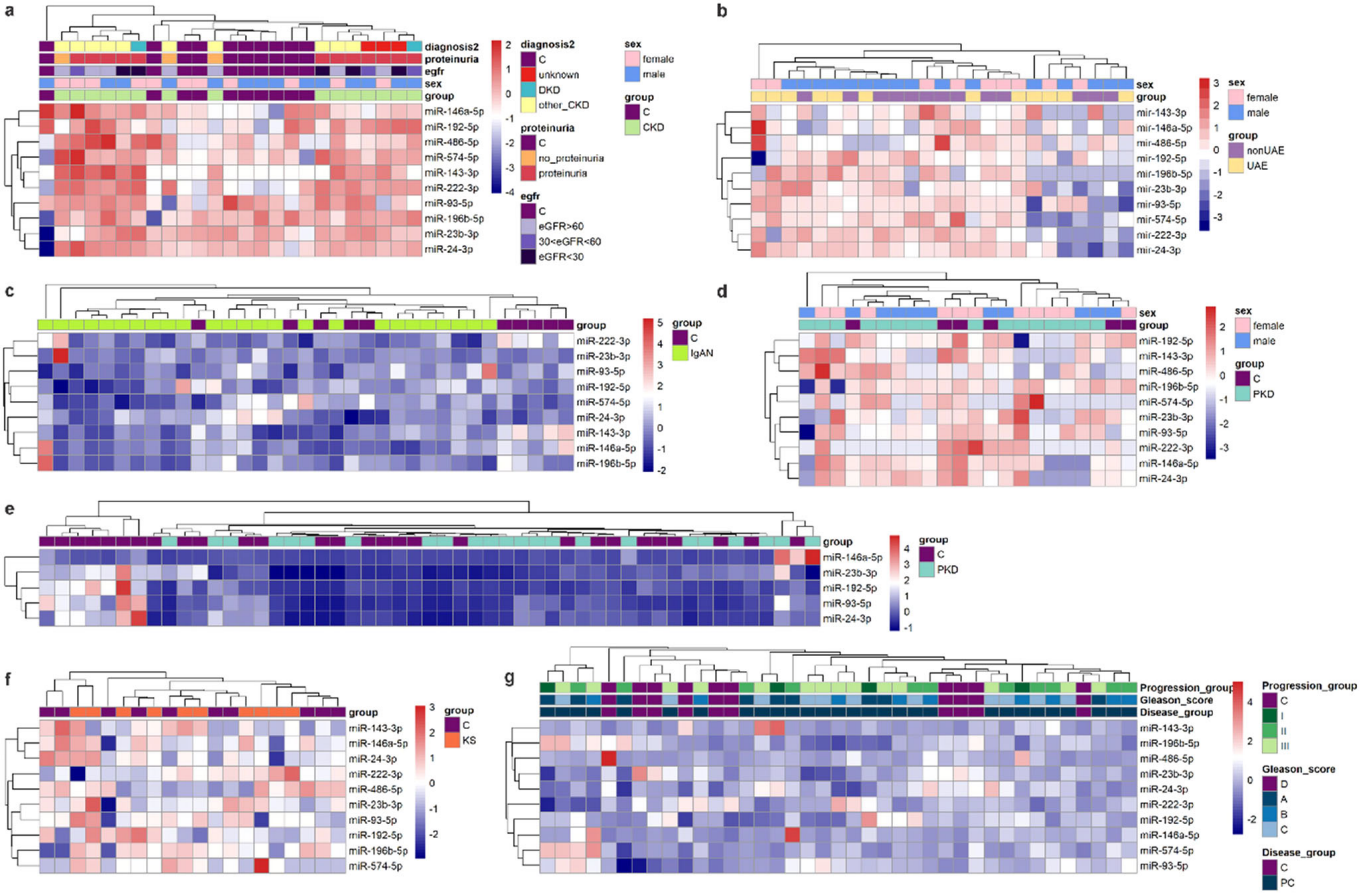
Heatmaps with hierarchical clustering of control published uEV datasets based on the VST normalized read counts of the 11 DE miRNAs in the T1D discovery cohort. (a) CKD, Khurana et al. 2017, (b) Hypertension, Perez‐Hernandez et al. 2021, (c) IgAN, Shankar et al. 2023, (d) PKD, Magayr et al. 2020, (e) PKD, Ali et al. 2024, (f) Kidney stones, Zhu et al. 2024, (g) Prostate cancer, Puhka et al. 2022. Chronic kidney disease (CKD), control (C), diabetic kidney disease (DKD), estimated glomerular filtration rate (eGFR), Immunoglobulin A nephropathy (IgAN), kidney stones (KS), no urinary albumin excretion (non‐UAE), polycystic kidney disease (PKD), prostate cancer (PC), with urinary albumin excretion (UAE).

To assess whether the DE miRNAs show specificity for DKD, we studied seven published control studies, including proteinuric and non‐proteinuric kidney diseases, that is, chronic kidney diseases of diverse aetiology, IgA and hypertensive nephropathy, polycystic kidney diseases, kidney stones and a prostate cancer dataset as a non‐kidney disease control (Figure 1, Table 6). Despite changes in some of the candidate miRNAs (Table 6), hierarchical clustering analysis using the group of 11 DE miRNAs from the discovery cohort (5 to 10 miRNAs were detected in the different datasets) showed a lack of clear clusters in any disease (Figure 7). However, we identified 2 clusters of proteinuric individuals in the CKD dataset.

Analyzing the samples separated by sex (in the datasets where sex was indicated), utilizing the DE miRNAs from the T1D male or female studies did not improve the clustering (Figure S6).

The replication studies thus supported changes in the candidate biomarker miRNAs in DKD and point out that the candidate markers show DKD specificity. (Table 6, Figures S5 and S6 and Figure 7)

### 3.7 Validation by qPCR

UEV miss stable reference miRNAs for qPCR‐based validation. We previously generated a candidate list of 14 stable uEV miRNAs (Barreiro et al. 2021) that we now evaluated further; we checked whether they were DE in the urine collection, diabetes study, DNAse I treatment and DKD sequencing analyses. Here, 5 candidate reference miRNAs remained stable in all the comparisons supporting their use as reference miRNAs (Table 7).

**TABLE 7:** Evaluation of 14 reference miRNA candidates across the preanalytical urine collection and T1D DKD male discovery and female replication datasets.

	ON vs. 24 h paired			FinnDiane vs. DIREVA			DNAse vs. non‐DNAse			Discovery: Normo vs. Macro			Discovery: ND vs. Macro			Replication: Normo vs. Micro + Macro		
miRNA	Avg. expr.	Log2FC	Padj.	Avg. expr.	Log2FC	Padj.	Avg. expr.	Log2FC	Padj.	Avg. expr.	Log2FC	Padj.	Avg. expr.	Log2FC	Padj.	Avg. expr.	Log2FC	Padj.
**let‐7a‐5p**	59077	0.11	1	79242	0.04	0.936	108884	0.20	1	79242	0.21	0.326	72574	0.43	0.449	162261	−0.19	0.327
let‐7b‐5p	28064	0.01	1	38027	−0.07	0.823	35216	0.29	1	38027	0.24	**0.044**	35409	0.18	0.922	61548	0.09	0.737
**let‐7c‐5p**	7836	−0.12	1	12224	0.22	0.411	14916	0.08	1	12224	0.05	0.868	11269	0.06	1	20083	−0.28	0.138
let‐7d‐5p	553	−0.34	1	887	0.12	0.668	2294	0.10	1	887	0.27	0.085	805	0.66	**0.026**	3468	0	0.992
let‐7f‐5p	31157	0.14	1	48044	0.24	**0.004**	118307	0.08	1	48044	0.03	0.868	43897	0.22	0.585	187186	−0.18	0.519
miR‐10a‐5p	124872	0.30	1	145276	−0.72	**0**	68854	−0.11	1	145276	−0.70	**0.006**	140772	−0.69	0.251	112657	−0.32	0.068
miR‐10b‐5p	664448	0.34	0.464	680834	−0.77	**0**	299072	−0.20	1	680834	−0.46	**0.044**	655759	−0.44	0.692	454460	−0.30	**0.045**
miR‐200b‐3p	751	−0.72	**0.021**	1452	0.54	**0.001**	665	0.13	1	1452	−0.10	0.754	1344	−0.30	0.872	7849	−0.05	0.971
**miR‐200c‐3p**	1496	−0.12	1	2485	0.22	0.274	4421	−0.21	1	2485	−0.04	0.868	2308	−0.13	1	6235	0.06	0.945
**miR‐204‐5p**	9439	0.08	1	11423	−0.17	0.603	9036	−0.24	1	11423	−0.25	0.294	10699	−0.23	1	37645	−0.29	0.058
miR‐23b‐3p	123	0.36	1	180	0.38	0.205	256	0.36	1	180	0.66	**0.017**	166	0.41	0.941	543	0.23	0.661
miR‐24‐3p	237	−0.54	1	397	0.31	0.488	1970	0.24	1	397	0.61	**0.050**	356	1.02	0.124	3530	0.13	0.709
miR‐26a‐5p	9476	0.03	1	14509	0.22	0.039	31124	−0.07	1	14509	0.02	0.868	13417	−0.02	1	48286	−0.18	0.456
**miR‐30c‐5p**	4659	−0.12	1	7001	0.16	0.464	5210	−0.12	1	7001	0.09	0.699	6375	0.38	0.490	42209	−0.07	0.895

*Note*: Five miRNAs (in bold) remained stable, and nineshowed differential expression in some of the comparisons (Padj. values <0.05 are highlighted in bold). Fold change (FC), macroalbuminuria (macro), microalbuminuria (micro), non‐diabetic (ND), normoalbuminuria (Normo), overnight (ON), p‐adjusted value (padj.).

**FIGURE 8:**
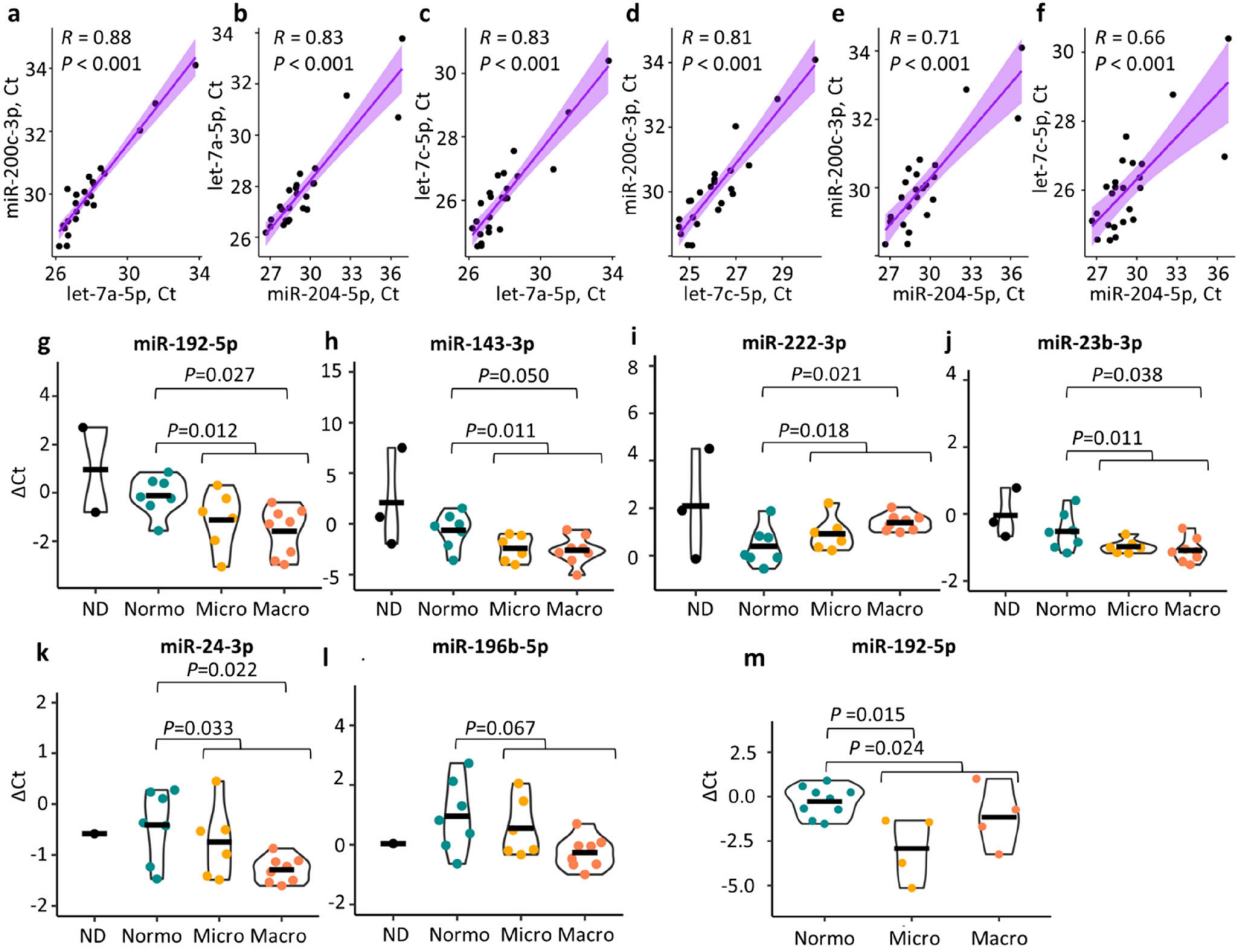
Quantitative PCR validation of selected miRNAs in the T1D cohorts. A subset of 21 individuals with T1D (*n* = 7 normo‐, 6 micro‐, and 8 macroalbuminuria) and 3 non‐diabetic individuals were included from the T1D male discovery cohort. (a and f) Spearman correlation of the cycle threshold (Ct) values between the candidate normalization miRNAs. (g–l) Delta Ct data for the candidate biomarker miRNAs represented in violin plots. The black horizontal line shows the mean value. (m) Quantitative PCR of miR‐192‐5p for 17 individuals from the T1D female replication cohort (*n* = 9 normo‐ vs. 4 micro‐ and 4 macroalbuminuria combined). Delta Ct values were calculated relative to the geometric mean of let‐7a‐5p and miR‐200c‐3p. MiRNAs were considered significantly differentially expressed with *p* values <0.05. Macroalbuminuria (Macro), microalbuminuria (Micro), non‐diabetic control (ND) normoalbuminuria (Normo).

We confirmed 4 candidate reference miRNAs by qPCR in a subset of the T1D discovery cohort (*n* = 21). Let‐7a‐5p and miR‐200c‐3p gave the best correlation between ct values, that is, similar stability, and showed no significant difference with and without DNAse treatment in the sequencing data (Figure S2c). They were thus used for normalization of 6 DE miRNAs in qPCR (Figure 8). To ensure the working range of the assays, the primers were tested with 1:10 cDNA dilution series (Figure S7). All the primers produced linear values, but the amplification efficiency was suboptimal for miR‐143‐3p and systematically too high for miR‐574‐5p. We thus excluded the miR‐574‐5p assay results.

In the T1D discovery cohort, qPCR confirmed a significant difference for the macro‐ (*n* = 8) versus normoalbuminuria (*n* = 7) and albuminuria (*n* = 14) versus normoalbuminuria groups for miR‐143‐3p, miR‐192‐5p, miR‐222‐3p, miR‐23b‐3p and miR‐24‐3p (Figure 8g–k, Table S6a,b). For the T1D replication cohort, miR‐192‐5p showed significant differences between the albuminuria (*n* = 8, 4 micro and 4 macroalbuminuria) and normoalbuminuria (*n* = 9) groups (Figure 8m, Table S6c,d).

### 3.8 Association and ROC Curve Analysis

**FIGURE 9:**
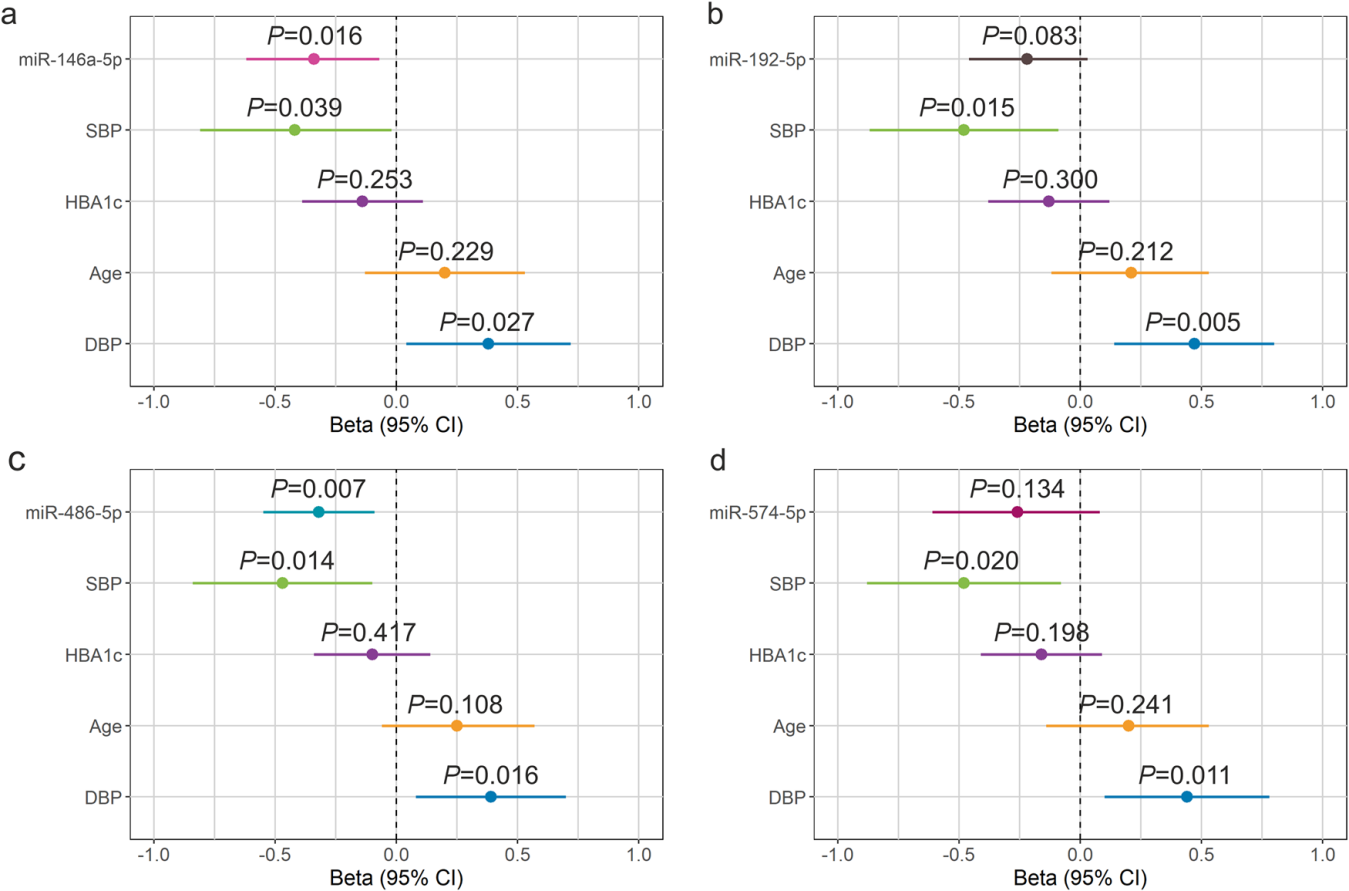
Association of the four differentially expressed miRNAs with eGFR decline (slope) in the T1D male discovery and female replication cohorts. (a) miR‐146a‐5p (*n* = 65). (b) miR‐192‐5p (*n* = 66). (c) miR‐486‐5p (*n* = 66). (d) miR‐574‐5p (*n* = 62). In (a–d), the model included the miRNA and age, DBP, HbA1C, and SBP. Diastolic blood pressure (DBP), estimated glomerular filtration rate (eGFR), glycated haemoglobin (HbA1c) and systolic blood pressure (SBP). All variables were converted to standard units.

Kidney dysfunction can be measured by eGFR decline, but measurement of a slope requires a long time for robust estimates—faster measurements of kidney function would be needed. As the correlation analysis had suggested that the miRNAs correlated with the eGFR slope, we further inspected this association through linear regression models for the 4 miRNAs (miR‐146a‐5p, miR‐192‐5p, miR‐486‐5p, miR‐574‐5p) validated in the T1D replication cohort and known clinical variables in DKD (age, HbA1c, SBP and DPB). The eGFR slope and/or clinical variables were not available for all samples; thus, we did the analysis for the combined T1D male discovery and female replication cohorts to achieve better statistical power (*n* = 66, 43 normo‐, 12 micro‐, and 11 macroalbuminuria). The miR‐146a‐5p and miR‐486‐5p were significantly associated with eGFR decline after adjusting for the other known clinical parameters (*p* < 0.05 and 0.01, respectively) (Figure 9).

**FIGURE 10:**
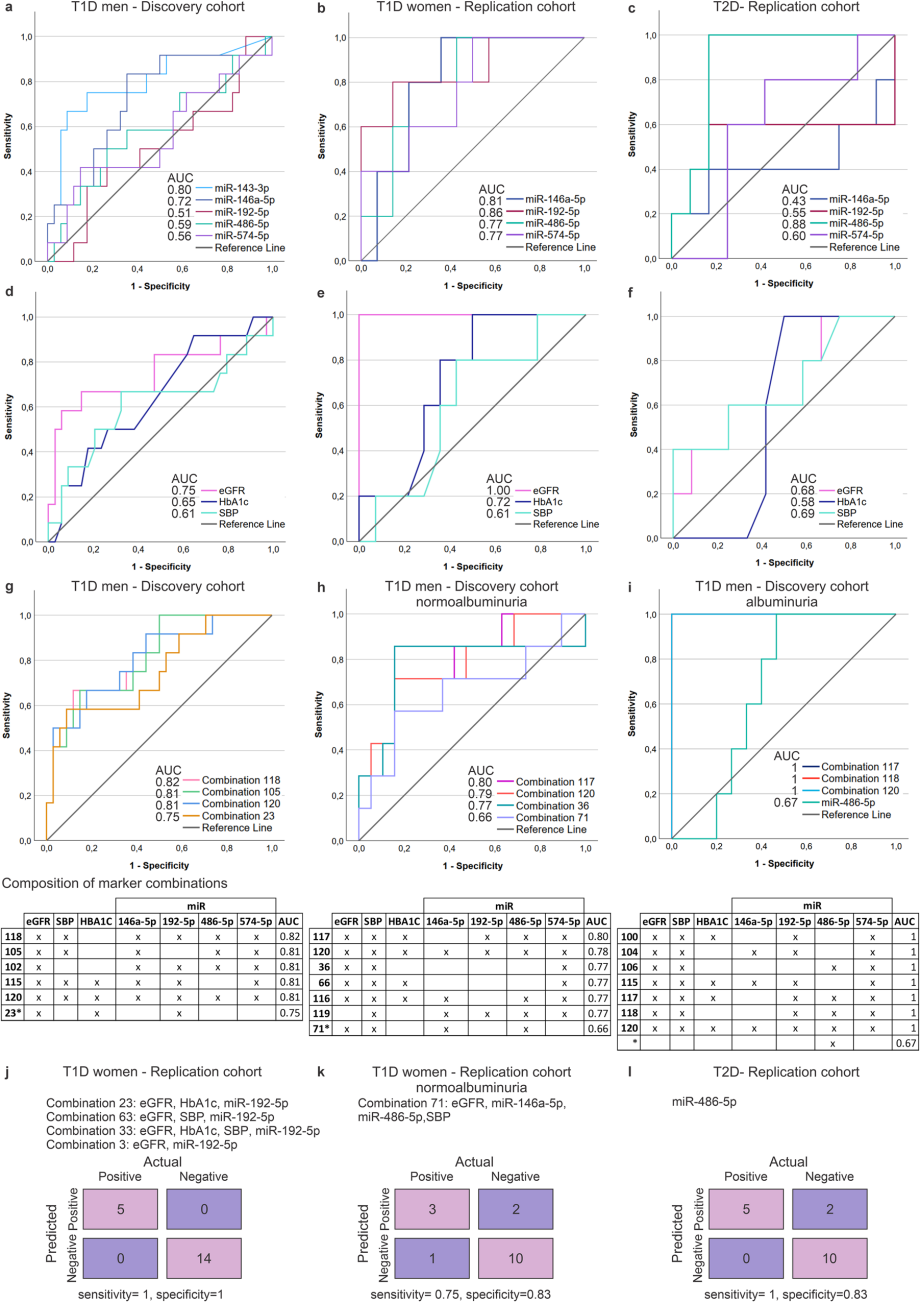
ROC (receiver operator characteristic) analysis and confusion charts using sequencing and clinical data. The number of individuals with available eGFR decline (slope) data has been indicated for each cohort (a–f) ROC curves of selected miRNAs and clinical measurements in discovery and replication cohorts for predicting eGFR decline (lowest quartile, Q1) of (a and d) ≤‐1.59 mL/min/1.73 m^2^/year for T1D male discovery cohort (*n* = 36, 16 normo‐, 10 micro‐, and 10 macroalbuminuria), (b and e) ≤‐1.66 mL/min/1.73 m^2^/year for T1D female replication cohort (*n* = 19, 16 normo‐, 2 micro‐, and 1 macroalbuminuria) and (c and f) ≤‐3.08 mL/min/1.73 m^2^/year for T2D replication cohort (*n* = 17, 10 micro‐, and 7 macroalbuminuria). (g–i) ROC curves of selected combinations of validated miRNAs in T1D cohorts (miR‐146a‐5p, miR‐192‐5p, miR‐486‐5p, miR‐574‐5p) and clinical measurements computed using CombiROC R package for predicting eGFR decline (lowest quartile Q1) of (g) same as in a,d, (h) ≤‐1.36 mL/min/1.73 m^2^/year for normoalbuminuric subgroup of T1D discovery cohort and (i) ≤‐2.46 mL/min/1.73 m^2^/year for albuminuric subgroup of T1D discovery cohort. (j–l) Results of the best classifications of replication cohorts using the models generated with CombiROC for predicting eGFR decline (lowest quartile, Q1) of (j) same as b,e (combination 23), (k) ≤‐1.18 mL/min/1.73 m^2^/year for normoalbuminuric subgroup of T1D replication cohort (combination 71) and (l) same as c,f (miR‐486‐5p). The compositions of the best combinations are disclosed in the tables, and all compositions are in Table S7. Area under the curve (AUC), Estimated glomerular filtration rate (eGFR, baseline eGFR was used as 1/eGFR), glycated haemoglobin (HbA1c), systolic blood pressure (SBP), type 1 diabetes (T1D), type 2 diabetes (T2D). * marker combinations that were not the best performing for the discovery cohort sample classification but were the best performing for the replication cohorts.

To assess whether the candidate miRNAs could be used to identify the fastest eGFR decliners (the lowest quartile, Q1) in early or late disease, we performed ROC analysis. We first studied individual miRNAs in comparison to some typical clinical measurements (eGFR—expressed as 1/eGFR, HbA1c, SBP). We plotted the 4 miRNAs for all cohorts, and miR‐143‐3p (the highest FC and adjusted *p* value in the discovery cohort), for the discovery cohort. The best performing miRNAs (area under the curve, AUC > 0.7) were miR‐143‐3p and miR‐146a‐5p for the T1D discovery cohort (*n* = 36, 16 normo‐, 10 micro‐ and 10 macroalbuminuria), miR‐192‐5p and miR‐146a‐5p for the T1D replication cohort (*n* = 19, 16 normo‐, 2 micro‐ and 1 macroalbuminuria) and miR‐486‐5p for the T2D replication cohort (*n* = 17, 10 micro‐ and 7 macroalbuminuria) (Figure 10a–c). Clinical measurements presented generally lower AUC values than the best performing miRNAs (Figure 10d–f), except for eGFR, which produced an AUC of 1 for the T1D replication cohort (Figure 10e).

The miRNAs and clinical measurements were next combined to evaluate whether ROC results could be improved and to generate models to test the classification of the replication cohorts. For this combinatorial analysis, we used the T1D discovery cohort (our largest cohort) for 3 analyses to classify the fastest eGFR decliners, including (i) all the samples, (ii) the normoalbuminuria subgroup, and (iii) the albuminuria (micro‐ and macroalbuminuria) subgroup (Figure 10g–i, Table S7a–c). For all 3 analyses, we found combinations with AUC ≥0.80.

To identify the best models to classify the fastest eGFR decliners in the replication cohorts, we selected from each combinatorial and individual miRNA analysis (i, ii, iii) the models with an AUC>0.65 and applied those models to the replication cohorts (Table S8a–c). The models with the best specificity and sensitivity are shown in Figure 10j–l. Altogether, with 0–2 false positives or negatives, the models showed the potential of uEV miRNAs for improved evaluation of kidney function from a single urine sample.

### 3.9 Target Pathway, mRNA and Protein Analysis

**FIGURE 11:**
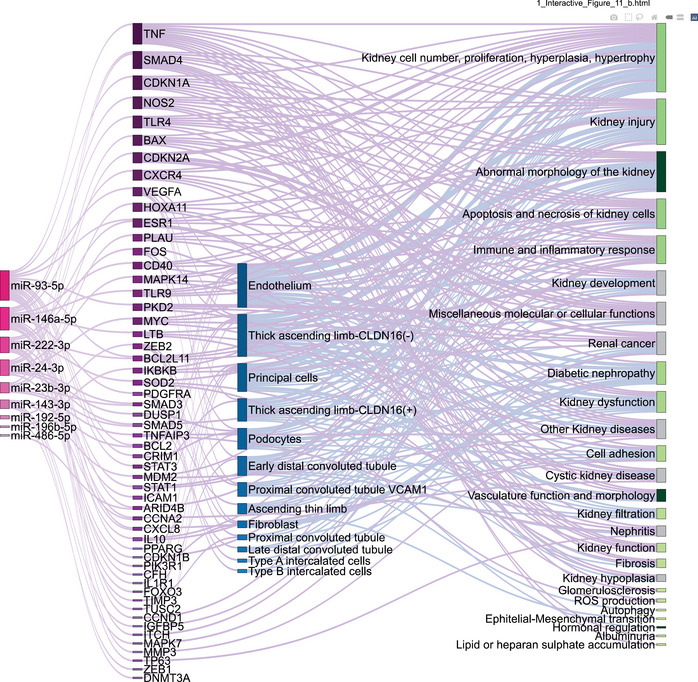
Interactive Sankey diagram depicting differentially expressed miRNAs between macro‐ and normoalbuminuria groups in the T1D discovery cohort, their experimentally validated target mRNAs, cell types where the targets have been found to be differentially expressed in kidney biopsies from T2D and DKD (scRNA seq data, Wilson et al. 2022) and disease or function associated with the target mRNAs. Light green nodes are associated with DKD, dark green nodes are associated with DKD but also with physiological conditions, and grey nodes are not associated with DKD. Source and target nodes are also disclosed in Table S11. Diabetic kidney disease (DKD), reactive oxygen species (ROS), RNA single‐cell sequencing (scRNA), type 1 diabetes (T1D) and type 2 diabetes (T2D).

To understand which pathways the DE miRNAs modulate in DKD, we studied mRNA targets expressed in the kidney (Table S9)—9 of the miRNAs had 54 experimentally validated mRNA targets. To simplify their target pathways, we grouped the various diseases and functions into larger categories (Table S10). From the 26 categories, 16 were directly DKD‐associated. (Figure 11). The top DKD‐associated categories (as judged by the number of incoming links/regulations) included kidney hypertrophy; kidney injury; apoptosis; and immune and inflammatory response. Other strongly DKD‐associated categories—such as glomerulosclerosis, albuminuria, fibrosis, and reactive oxygen species (ROS) production—were represented but with less mRNA targets. To obtain more evidence for the relevance of the targeted mRNAs and pathways in DKD, we incorporated kidney single cell sequencing data from individuals with T2D and DKD (Wilson et al. 2022). We identified 19 cell‐type categorized mRNA targets that were DE in DKD biopsies. The cells with most of the dysregulated mRNAs (number of incoming links) were endothelial cells, thick ascending limb of Henle's loop CLDN16 (‐), principal cells, podocytes, and fibroblast.

Additionally, a predicted target search showed many other interesting targets linked to DKD, for example, transcription factors HNF1A, HNF1B, HNF4A, SMAD2 and STAT6 (Table S9).

We further studied whether the proteins coded by the 54 target mRNAs (see Figure 9) or by the DE uEV mRNAs (including the stress score) we identified in DKD (Dwivedi et al. 2023), were dysregulated in individuals with T1D and T2D. We had a large plasma proteomics dataset available in the UK Biobank that allowed comparisons of individuals with and without kidney or ophthalmic diabetes complications. Males and females were analyzed both together and separately when possible. Data were available for 34 proteins. Logistic regression model analysis showed a statistically significant association (Bonferroni Padj <0.05) with kidney complications for 9 proteins ‐ 3 were in common with ophthalmic complications ‐ in the combined and male cohorts (Table 8). In summary, all target analyses supported the relevance of the miRNA candidates in DKD.

**TABLE 8:** Statistically significant associations from the UK biobank plasma proteomic analysis using logistic regression models.

Protein	miRNA	Sex	Case	Control	*n* cases	*n* controls	*β*	SEM	*z*‐score	Padj.
BCL2	miR‐143‐3p, miR‐93‐5p, miR‐23b‐3p*	Combined	T2D KC	T2D	85	3822	0.31	0.06	4.83	1.41E‐04
BCL2	miR‐143‐3p, miR‐93‐5p, miR‐23b‐3p*	Males	T2D KC	T2D	60	2271	0.31	0.08	3.92	9.22E‐03
BCL2L11	miR‐222‐3p, miR‐93‐5p, miR‐24‐3p*	Males	T2D KC	T2D	60	2271	0.64	0.17	3.83	1.29E‐02
BCL2L11	miR‐222‐3p, miR‐93‐5p, miR‐24‐3p*	Combined	T2D KC	T2D	85	3822	0.48	0.14	3.52	4.44E‐02
BCL2L11	miR‐222‐3p, miR‐93‐5p, miR‐24‐3p*	Combined	T2D OC	T2D	435	3510	0.22	0.06	3.84	1.26E‐02
CRIM1	miR‐93‐5p	Combined	T2D KC	T2D	85	3822	0.54	0.08	7.07	1.64E‐10
CRIM1	miR‐93‐5p	Males	T2D KC	T2D	60	2271	0.67	0.10	7.01	2.44E‐10
IL1R1	miR‐146a‐5p	Combined	T2D KC	T2D	85	3822	0.55	0.08	7.14	9.51E‐11
IL1R1	miR‐146a‐5p	Males	T2D KC	T2D	60	2271	0.62	0.09	6.89	5.71E‐10
IL1R1	miR‐146a‐5p	Combined	T2D OC	T2D	435	3510	0.35	0.04	8.75	2.22E‐16
IL1R1	miR‐146a‐5p	Males	T2D OC	T2D	283	2067	0.34	0.05	6.94	4.03E‐10
IL1R1	miR‐146a‐5p	Females	T2D OC	T2D	152	1443	0.36	0.07	5.36	8.61E‐06
MMP3	miR‐93‐5p, miR‐574‐5p*	Combined	T2D KC	T2D	85	3822	0.55	0.10	5.48	4.30E‐06
MMP3	miR‐93‐5p, miR‐574‐5p*	Males	T2D KC	T2D	60	2271	0.66	0.12	5.34	9.69E‐06
PDGFRA	miR‐146a‐5p, miR‐24‐3p*, miR‐93‐5p*	Combined	T1D KC	T1D	12	416	0.83	0.23	3.65	8.89E‐03
PDGFRA	miR‐146a‐5p; miR‐24‐3p*, miR‐93‐5p*	Combined	T2D KC	T2D	85	3822	0.59	0.08	7.19	6.77E‐11
PDGFRA	miR‐146a‐5p, miR‐24‐3p*, miR‐93‐5p*	Males	T2D KC	T2D	60	2271	0.65	0.10	6.65	3.10E‐09
PDGFRA	miR‐146a‐5p, miR‐24‐3p*, miR‐93‐5p*	Combined	T1D OC	T1D	90	354	0.46	0.11	4.41	1.07E‐03
PDGFRA	miR‐146a‐5p, miR‐24‐3p*, miR‐93‐5p*	Males	T1D OC	T1D	48	200	0.66	0.15	4.39	1.15E‐03
PDGFRA	miR‐146a‐5p, miR‐24‐3p*, miR‐93‐5p*	Combined	T2D OC	T2D	435	3510	0.17	0.05	3.80	1.51E‐02
RBP5	miR‐192‐5p*, miR‐574‐5p*	Males	T2D KC	T2D	60	2271	0.51	0.11	4.54	5.86E‐04
RBP5	miR‐192‐5p*, miR‐574‐5p*	Combined	T2D KC	T2D	85	3822	0.40	0.09	4.32	1.58E‐03
TNF	miR‐93‐5p, miR‐24‐3p*	Combined	T2D KC	T2D	85	3822	0.38	0.06	6.65	2.96E‐09
TNF	miR‐93‐5p, miR‐24‐3p*	Males	T2D KC	T2D	60	2271	0.43	0.07	5.86	4.69E‐07
VEGFA	miR‐93‐5p	Males	T2D KC	T2D	60	2271	0.57	0.12	4.80	1.65E‐04
VEGFA	miR‐93‐5p	Combined	T2D KC	T2D	85	3822	0.46	0.10	4.57	4.90E‐04

*Note*: *miRNAs predicted with high or moderate confidence (Table S9)—miRNAs without stars have targets with experimental evidence, kidney complications (KC), ophthalmic complications (OC), p‐adjusted value (padj.), standard error (SEM), type 1 diabetes (T1D), type 2 diabetes (T2D).

## 4 Discussion

The uEV provide a view of the kidney tissue, which is rarely biopsied in individuals with diabetes. However, relatively few studies have profiled uEV miRNAs in DKD. Common shortcomings regarding the uEV miRNA studies are poor characterization of the preanalytical variables and uEV isolates, low statistical power, heterogenous groups, and lack of replication in independent cohorts. Here, we used 16 multinational well characterized T1D, T2D and control cohorts to evaluate potential uEV‐miRNA changes in DKD, excluding changes due to preanalytical factors. We found candidate DKD marker miRNAs, which regulate diverse DKD‐associated mRNAs, proteins and pathways and could classify the individuals with the fastest eGFR decline in early to late DKD.

MiRNA biomarker candidates tend to differ between studies, and not least due to preanalytics (Barreiro et al. 2023; Erdbrügger et al. 2021; López‐Guerrero et al. 2023). We therefore started by exploring our preanalytical variables, which revealed some DE miRNAs between the overnight versus 24 h urine collections. We then selected only the unaffected miRNAs for further analysis of the DE and reference miRNAs—the safest strategy when including different cohorts in the same study.

Pathway analysis of the DE miRNAs revealed their overall relevance in DKD: many target mRNAs have been linked to DKD (Fu et al. 2015; Kato et al. 2011; Kato et al. 2007; Yang et al. 2012; Yu et al. 2023) and they are part of the classical DKD pathways (DeFronzo et al. 2021; Thomas et al. 2015; Tuttle et al. 2022). Interestingly, judged by the number of targets per disease pathway, uEV captured more changes at the early DKD stage, such as kidney hypertrophy, kidney injury, and changes in the kidney morphology for example, expansion of mesangial matrix (Anders et al. 2018; Lin et al. 2018). Other hallmark pathways, such as autophagy and ROS production (DeFronzo et al. 2021), and advanced DKD changes like glomerulosclerosis and fibrosis (Anders et al. 2018; Lin et al. 2018), were less targeted by the DE miRNAs. Finding of relatively more early pathways might be because our discovery study included less individuals at late DKD stages. Incorporation of single cell sequencing data from human kidney biopsies with DKD (Wilson et al. 2022) showed that many of the target‐mRNAs were DE in the hallmark kidney cell types of DKD, such as the endothelium, podocytes, and proximal tubule cells (Anders et al. 2018). Since the biopsies were taken at the early stages of DKD (Wilson et al. 2022), it is possible that the DE biopsy mRNAs targeted by the DE uEV miRNAs and the final regulated pathways all relate to the early changes in DKD. Additionally, exploration of the proteins encoded by the DE miRNAs’ target mRNAs and the stress score mRNAs revealed nine proteins associated with kidney and ophthalmic complications in independent T1D and T2D studies. Finding such a large proportion of target proteins (26%) changed in plasma and prior data of protein changes in DKD or CKD—for example, for plasma VEGF (Hovind et al. 2000), plasma IL1R1 (Niewczas et al. 2019), plasma and podocyte CRIM1 (Schlosser et al. 2024), tubular and podocyte BCL2 (C. Yang et al. 2023; Zhou et al. 2023), plasma and serum MMP3 (Peeters et al. 2015; Peeters et al. 2017), and plasma TNF (Kamei et al. 2018)—support the hypothesis that the DE miRNAs are important regulators in DKD.

The four validated miRNAs in DKD of T1D showed individually or in combinations a good performance in the association and ROC analyses and classifications for the T1D or T2D replication cohorts. From these candidate DKD markers, the miR‐192‐5p was upregulated in the discovery and many replication cohorts and correlated with the eGFR slope and the stress score. In line, prior studies have shown miR‐192‐5p upregulation in urine (Barreiro et al. 2023; Jia et al. 2016; Wan et al. 2023; Yang et al. 2017) and correlation between its expression in uEV and urinary sediments and the eGFR in individuals with T2D and DKD (X. Yang et al. 2017; Zapała et al. 2023). Early on, the miR‐192 was studied in human kidney cortical tissues and in glomeruli of rat and mouse models of DKD (Wan et al. 2023). The miR‐192 upregulation in the kidneys could link to for example, the inhibition of GLP‐1 receptor or SMAD/TGF‐β and PTEN/PI3K/AKT signalling pathways that regulate epithelial‐to‐mesenchymal transition and fibrosis (Wan et al. 2023). Interestingly, Chatterjee and colleagues (Chatterjee et al. 2023) reported that miR‐192‐5p, miR‐146a‐5p and miR‐21‐5p—targeting the SMAD/TGF‐β pathway—were changed in blood EVs from individuals with the cardiovascular‐kidney‐metabolic syndrome. Treating proximal tubule cells on a chip with these plasma EVs induced kidney injury markers. As the exact same miRNAs were changed in our DKD study (even if miR‐21‐5p was excluded due to changes in the preanalytical study), it suggests that for diseases with cardiorenal complications, kidney injury coincides with the secretion of blood and uEV carrying these miRNAs.

The association of miR‐146a‐5p with diabetes complications has been proven by many studies (Ghaffari et al. 2023). However, the reports have been contradictory for example, with tissue up‐ or downregulation and pro‐ or anti‐inflammatory roles in human T2D kidney biopsies and rodent diabetes models (Bhatt et al. 2016; Huang et al. 2014; Lee et al. 2017). Furthermore, uEV miR‐146a‐5p was found to be upregulated in lupus nephritis (Perez‐Hernandez et al. 2021), polycystic kidney disease (Ali et al. 2024b), bladder cancer (Prieto‐Vila et al. 2022) and prostate cancer (Puhka et al. 2022). Studies on uEV cargo sorting are ongoing and could apply active or passive mechanisms serving for (disease‐specific) cargo functions or disposal (Dellar et al. 2022; O'Grady et al. 2022). MiRNAs are also versatile (post‐) transcriptional regulators (Catalanotto et al. 2016; Bachurski et al. 2019; Selbach et al. 2008), and known as repressors, or rarely, activators of translation (Vasudevan et al. 2007). Consequently, particular miRNAs could be dysregulated in diverse diseases. Nevertheless, we showed that combinations of the DE miRNAs from our DKD discovery study—including the miR‐146a‐5p—did not separate the control study cohorts, including proteinuric kidney diseases of hypertensive and IgA nephropathy or PKD, kidney stones and prostate cancer. The DE miRNAs did separate the CKD study groups based on proteinuria to some extent, which might reflect the underlying similarity of DKD and CKD and of the study designs. Further, our analysis of the published DKD datasets supported changes in 10 DE miRNAs. When searching for replication datasets, we encountered difficulties related to data availability and reporting. It is plausible that the different study designs of the published datasets and different EV methods could explain why some miRNAs showed smaller changes than in our data. Taken together, a larger combination of miRNAs has the potential to provide DKD‐specificity.

Mir‐574‐5p has been linked to immune and inflammatory responses, for example, its overexpression in DKD downregulated C7 in mesangial cells (Guo et al. 2021). The DKD‐association of miR‐486‐5p is suggested by its downregulation in glomeruli and proximal tubule cells of biopsies from individuals with DKD (Baker et al. 2017) and in mesangial cells exposed to high glucose, which promotes fibrosis (Duan et al. 2021). Interestingly, in our DNAse testing, the miR‐486‐5p, unlike other miRNAs, showed some sensitivity to DNAse. MiR‐486‐5p is the most abundant miRNA in plasma (Bai et al. 2019) and this opens the question whether the increased uEV miR‐486‐5p in DKD could originate from the blood. In healthy conditions, the kidney filtration barrier limits the passage from blood to urine, but when the barrier is disrupted, like in DKD, the passage might increase at least for some selected miRNAs (Erdbrügger et al. 2021; Neal et al. 2011; Pazourkova et al. 2016) leading to concomitant increase in blood miRNAs sticking to the uEV corona. As DNA has been shown to reside on the EV corona (Yerneni et al. 2022), a DNAse treatment of uEV could generally reduce the corona that is, remove both the DNA and miRNAs in the corona. However, same miR‐486‐5p changes between compared groups were observed in cohorts treated with or without DNAse. Thus, to our understanding, more evidence is needed to clarify the blood‐to‐urine leaking phenomenon and its magnitude.

We foresee different potential use of the validated miR‐146a‐5p, miR‐192‐5p, miR‐486‐5p and miR‐574. Because they all performed fine as part of miRNA and clinical measurement combinations to discriminate the fastest eGFR decliners in the T1D normoalbuminuria groups, they might possess the power to serve as early DKD biomarkers. They performed fine also for the T1D albuminuria groups suggesting further use as markers at late stages. In contrast, in the T2D cohort, only the miR‐486‐5p performed satisfactorily as a marker for late stage. The difference between the T1D and T2D cohorts was expected based on the different disease aetiologies, T2D heterogeneity and co‐morbidities (Ahlqvist et al. 2018; Anders et al. 2018; van Raalte et al. 2024). However, in addition to miR‐486‐5p, changes in several other miRNAs were supported by the analysis of all the different replication datasets, thus some prominent mechanisms appear to be shared between the diabetes types 1 and 2.

Along with the common DE miRNAs of the T1D male discovery and female replication cohorts, we discovered some unique miRNAs. This interested us, because sex‐based differences in the disease mechanisms and course could affect the DKD biomarkers. As almost all the replication and control cohorts were male dominant (or male only), we considered potential sex‐differences by presenting the results with sex‐labels or sex‐stratified whenever data was available. Interestingly, two of the potential male‐biased miRNAs (not DE in the female only cohort), target HNF4A (miR‐143‐3p) or KDM6A (miR‐23b‐3p), which were recently linked to a sex‐specific course of DKD (Clotet‐Freixas et al. 2024). HNF4A, known from maturity‐onset diabetes of the young (MODY) type 1, regulates kidney metabolism (Yamagata, Furuta, et al. 1996) with androgen receptor particularly in the proximal tubule cells (Clotet‐Freixas et al. 2024; Piani et al. 2021; Pihlajamaa et al. 2014). KDM6A is a X‐linked gene and an epigenetic regulator of genes and metabolism in the kidneys and proximal tubule cells. Another potential male‐biased miRNA, miR‐24‐3p (Zhu et al. 2013) targets further MODY genes, HNF1A (Yamagata et al. 1996) and HNF1B (Hua Tan et al. 2022), which associate with the regulation of sodium/glucose cotransporter 2 in proximal tubule cells (Pontoglio et al. 2000) and kidney disease susceptibility (Hua Tan et al. 2022), respectively. It is noteworthy that our discovery of dysregulated uEV miRNAs targeting the MODY genes could suggest an unexpected connection between the common and rare forms of diabetes and DKD via epigenetic deregulation.

Similar to our results, earlier urine/uEV studies of T1D (Argyropoulos et al. 2015) observed miR‐23b‐3p and miR‐192‐5p changes in DKD including normoalbuminuric individuals subsequently developing microalbuminuria and differences between sexes. In our study, miR‐192‐5p classified the fastest eGFR decliners better in females, even if it was DE in both sexes. MiR‐222‐3p, a potential male‐biased miRNA, is X‐linked (Di Palo et al. 2020), which could partly explain its sex‐specific regulation in DKD. Finally, the plasma proteins targeted by the DE miRNAs (discovered in males) associated with DKD only in male and combined sex cohorts of the UK Biobank. In line, upregulation of plasma VEGF (target of miR‐93‐5p) was found earlier only in males with DKD (Hovind et al. 2000). Plasma/serum MMP3 (target of miR‐93‐5p, and miR‐574‐5p) was associated with eGFR decline (Peeters et al. 2017) or macroalbuminuria and diabetic retinopathy (Peeters et al. 2015) in both sexes. While the small size of our female T1D DKD replication and T2D kidney complication groups certainly affected the statistical power, the female T2D ophthalmic complication group was large. Since diabetic retinopathy is a strong predictor of DKD (Shi et al. 2023), some shared protein changes can be expected in both diagnoses. However, only IL1R1 (target of miR‐146a‐5p, DE in both sexes) showed changes in the female T2D ophthalmic complication group along with all male and combined groups. Put against this background, the DE miRNAs appear to target key metabolic and other kidney regulators thereby affecting DKD progression in sex‐specific and sex‐independent ways. For maximally specific or generally applicable DKD biomarkers, our results support the idea to study uEV both separately and together in males and females.

The strengths of our work include focus on T1D, high‐quality samples and characterization of the participants, a long follow‐up time, finding of miRNA candidates that predict eGFR loss independent of proteinuria, replication of the results in several independent cohorts and exploration in other kidney or prostate diseases. We present an experimental design—albuminuria groups and clinical characteristics—suited for the discovery of early but also later changes in DKD. We generated a unique insight into the technical/biological replicability and to disease, diabetes subtype, sex, mRNA, protein, pathway and kidney cell type specificity. Moreover, we characterized our uEV preparations (here and previously; [Barreiro et al. 2020; Barreiro et al. 2021; Dwivedi et al. 2023; Puhka et al. 2017; Puhka, Takatalo, et al. 2017]) with many current techniques following MISEV guidelines (Welsh et al. 2024). The sample number is high compared with previous uEV small‐RNA sequencing studies. However, we recognize that the study size is still small for a biomarker study and that in the case of the T1D female validation cohort, the number of individuals with albuminuria is not optimal. Thus, our results should be validated in larger and yet more varied cohorts.

In conclusion, we discovered non‐invasive candidate miRNA markers for DKD in individuals with T1D. The miRNAs hold the potential to serve as early biomarkers for DKD, because they can modulate early DKD pathways and classify T1D individuals with normoalbuminuria and fast eGFR decline. Our results support that combinations of miRNAs and clinical variables could increase the diagnostic power. Additionally, we advance the uEV liquid biopsy field by pinpointing candidate reference miRNAs and miRNAs affected by common pre‐analytical variables.

## Conflicts of Interest

Per‐Henrik Groop is an advisory board member for AstraZeneca, Bayer, Boehringer Ingelheim, Merck Sharp & Dohme, Nestlé, Novo Nordisk, and has received lecture fees from Astellas, AstraZeneca, Bayer, Berlin Chemie, Boehringer Ingelheim, Menarini, Merck Sharp & Dohme, Medscape, Novo Nordisk, and Sanofi. Andrea Ganna is founder of Real World Genetics Oy.

## Supporting information




**Supplementary Figure S1**: Transmission electron microscopy micrographs.
**Supplementary Figure S2**: Effect of DNAse I treatment on sequencing output and candidate miRNAs in four pairs of samples.
**Supplementary Figure S3**: Pairwise Spearman correlations between normalized miRNA counts in T1D discovery cohort and clinical measurements for all comparisons with absolute R value ≥0.3 and p<0.05.
**Supplementary Figure S4**: Heatmap clustering of the female T1D replication cohort using VST normalized read counts and the 11 differentially expressed miRNAs of the T1D male cohort.
**Supplementary Figure S5**: Replication of findings using published miRNA profiling datasets of uEV in DKD.
**Supplementary Figure S6**: Heatmap with hierarchical clustering of control published uEV datasets based on the VST normalized read counts of the DE miRNAs in the T1D cohorts with sex stratification.
**Supplementary Figure S7**: qPCR primer efficiency.

**Supplementary Table S1**: Statistical calculations related to clinical data Tables 2 and 3.

**Supplementary Table S2**: Differential expression analysis of preanalytical comparisons.

**Supplementary Table S3**: T1D male discovery cohort raw sequencing counts and differential expression analysis.

**Supplementary Table S4**: Differential expression analysis of T1D female replication cohort. DEseq2 results from analyzing albuminuria (microalbuminuria and macroalbuminuria) versus normoalbuminuria groups.

**Supplementary Table S5**: Differential expression analysis of T2D replication cohort (females and males). DEseq2 results from analyzing macroalbuminuria versus microalbuminuria groups.

**Supplementary Table S6**: Descriptive statistics, ANOVA results and multiple comparisons of qPCR data from T1D discovery and replication cohorts.

**Supplementary Table S7**: Combinatorial analysis of candidate miRNA markers (miR‐146a‐5p, miR‐192‐5p, miR‐486‐5p, miR‐574‐5p) and clinical measurements (eGFR, SBP, HBA1C) using CombiROC for the T1D male discovery cohort.

**Supplementary Table S8**: Classification of T1D and T2D replication cohorts using the models presented in Supplementary Table S7 (lowest quartile, Q1).

**Supplementary Table S9**: Predicted and experimentally observed mRNA targets for the differentially expressed miRNAs. Analysis done using ingenuity pathway analysis (Qiagen).

**Supplementary Table S10**: Grouping of the IPA diseases or functions into 26 categories to reduce the complexity of the interactive Sankey diagram.

**Supplementary Table S11**: Source and target nodes of the interactive Sankey diagram.

## Data Availability

The raw count data for miRNA sequencing data used in this manuscript (discovery phase—T1D FinnDiane and DIREVA studies) is provided in Table S3a. Individual‐level data for the study participants are not publicly available because of the restrictions due to the study consent provided by the participant at the time of data collection.
