# Supplementary material for: Selected miRNAs in Urinary Extracellular Vesicles Show Promise for Early and Specific Diagnostics of Diabetic Kidney Disease
Source: J Extracell Biol. 2025 Oct 14;4(10):e70089. doi: 10.1002/jex2.70089 (PMC12519432; doi:10.1002/jex2.70089)
Supplement: Supplementary file 1 — Supplementary Figure S1: Transmission electron microscopy micrographs. Supplementary Figure S2: Effect of DNAse I treatment on sequencing output and candidate miRNAs in four pairs of samples. Supplementary Figure S3: Pairwise Spearman correlations between normalized miRNA counts in T1D discovery cohort and clinical measurements for all comparisons with absolute R value ≥0.3 and p<0.05. Supplementary Figure S4: Heatmap clustering of the female T1D replication cohort using VST normalized read counts and the 11 differentially expressed miRNAs of the T1D male cohort. Supplementary Figure S5: Replication of findings using published miRNA profiling datasets of uEV in DKD. Supplementary Figure S6: Heatmap with hierarchical clustering of control published uEV datasets based on the VST normalized read counts of the DE miRNAs in the T1D cohorts with sex stratification. Supplementary Figure S7: qPCR primer efficiency. [file JEX2-4-e70089-s005.docx]

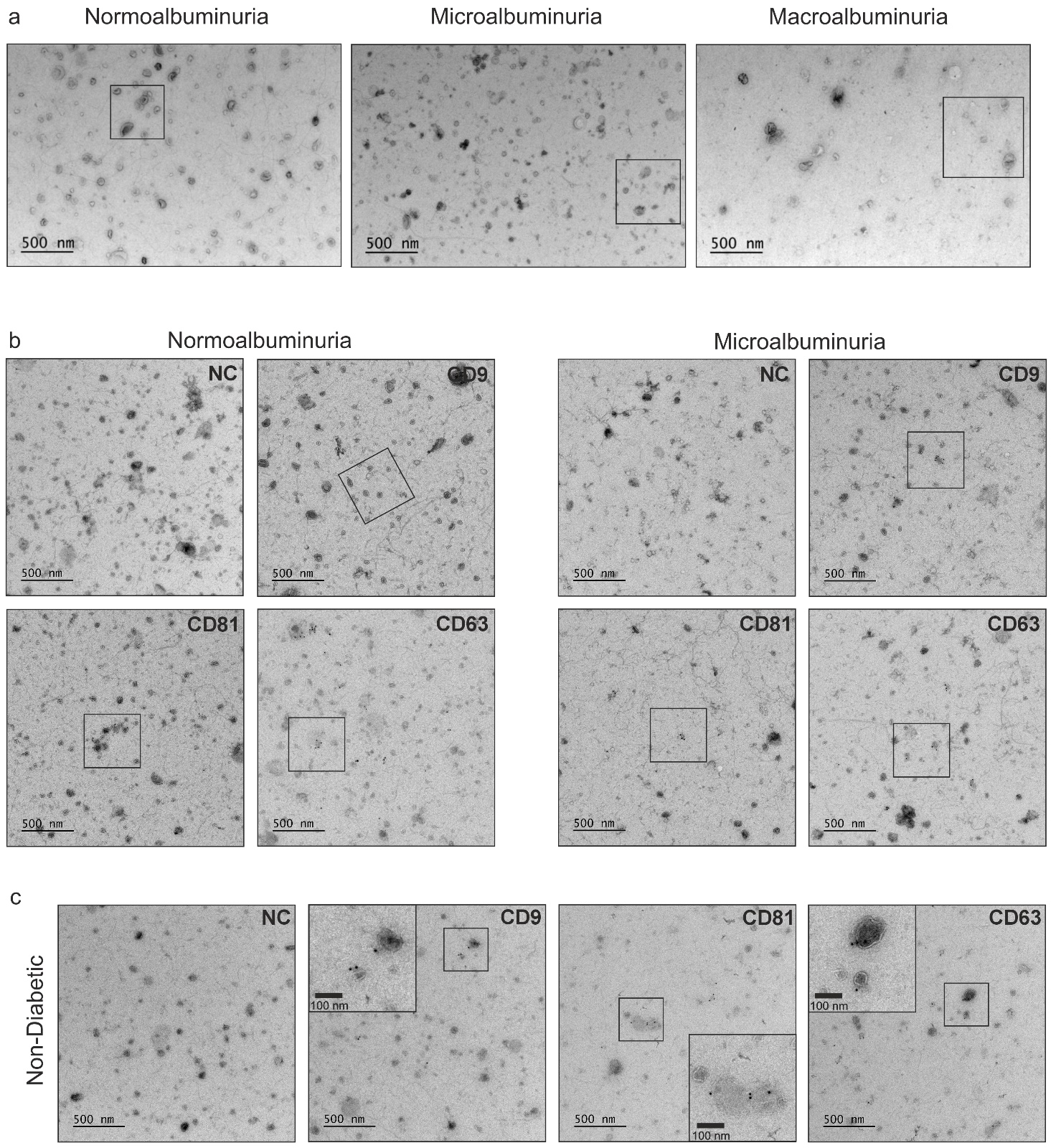


**Supplementary Figure S1:** Transmission electron microscopy micrographs. (a) Wide field micrographs from close ups showed in figure 2b. (b) Wide field micrographs from immunodetection of CD9, CD81 and CD63. Close ups are shown in figure 2c. (c) Wide field micrographs and close ups showing immunodetection of CD9, CD81 and CD63 in uEV derived from non-diabetic individuals. Negative control (NC).


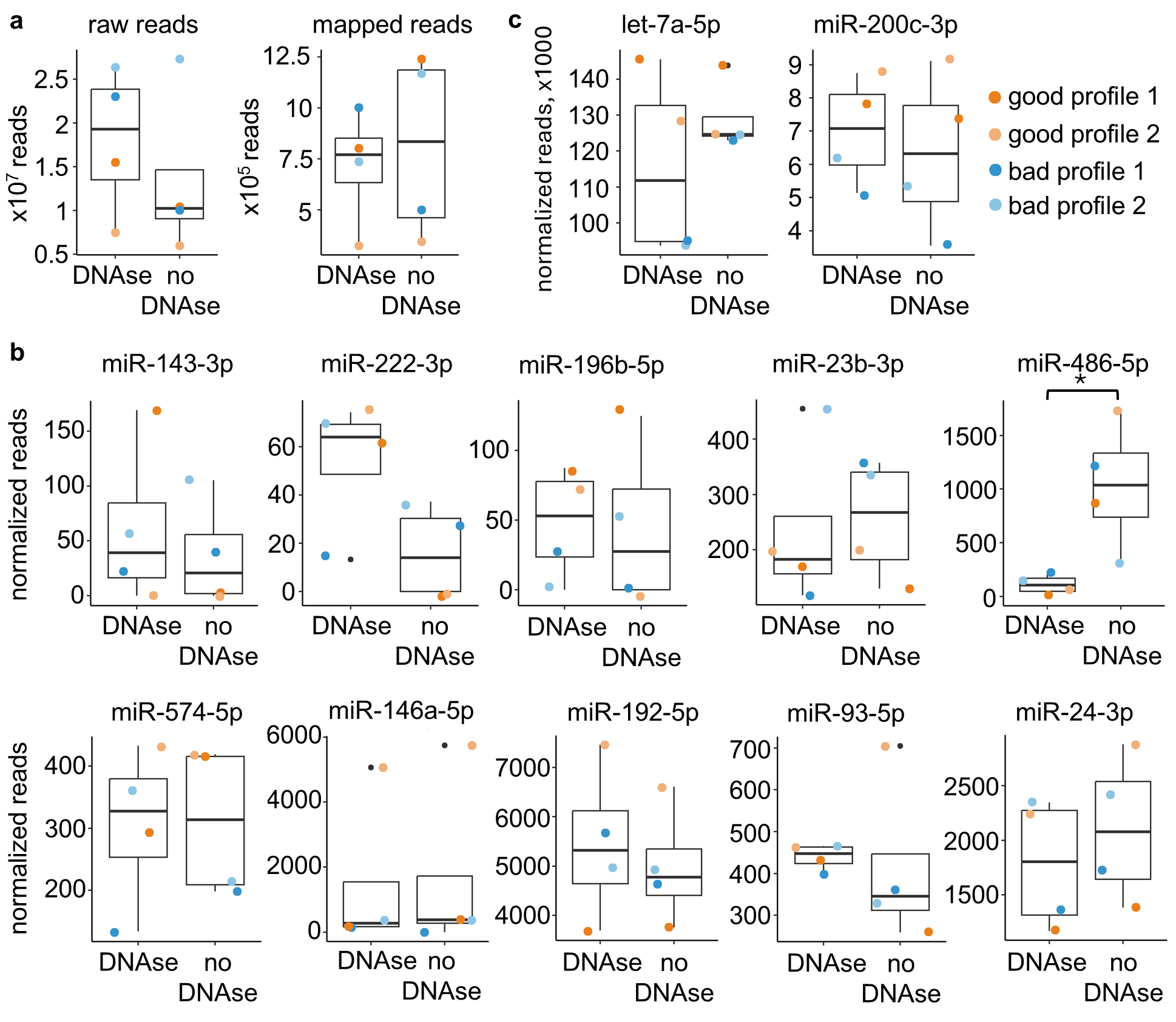


**Supplementary Figure S2:** Effect of DNAse I treatment on sequencing output and candidate miRNAs in four pairs of samples. Good and bad profiles refer to bioanalyzer pico chip electropherograms as described in (Dwivedi et al, 2023). (a) number of raw and mapped sequencing reads. (b) Discovery cohort differentially expressed miRNAs and (c) candidate reference miRNAs.


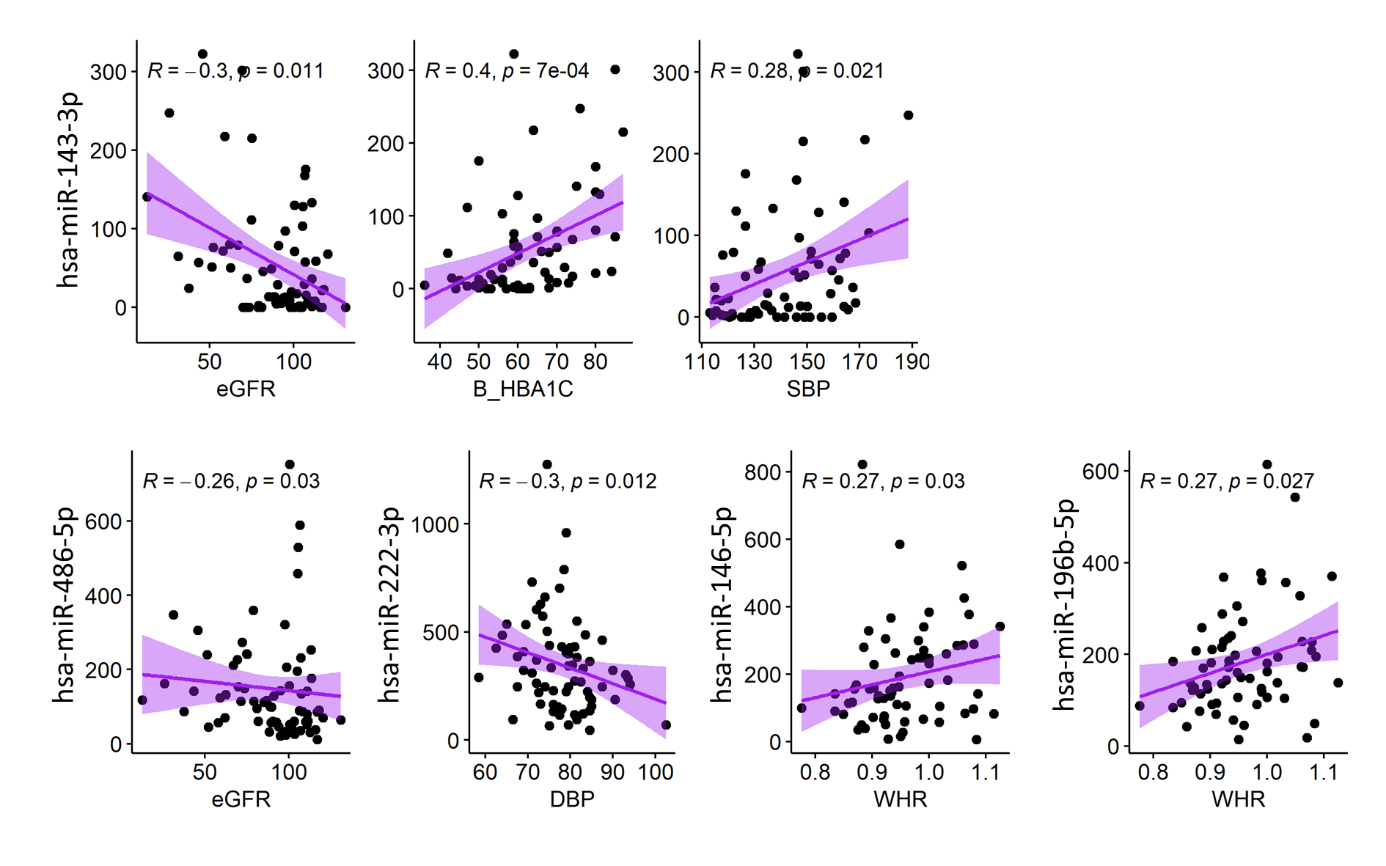
**Supplementary Figure S3:** Pairwise Spearman correlations between normalized miRNA counts in T1D discovery cohort and clinical measurements for all comparisons with absolute R value ≥0.3 and p<0.05. Diastolic blood pressure (DBP), estimated glomerular filtration rate (eGFR), glycated hemoglobin (HBA1C), systolic blood pressure (SBP), waist-hip ratio (WHR).


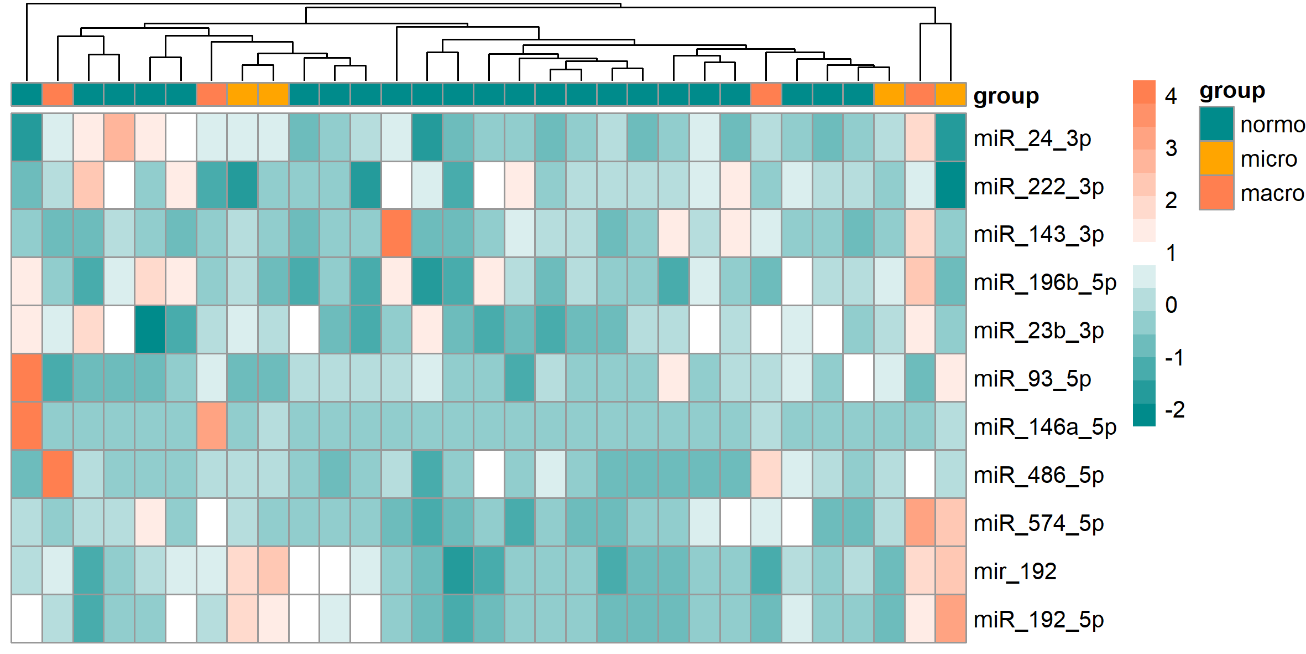


**Supplementary Figure S4:** Heatmap clustering of the female T1D replication cohort using VST normalized read counts and the 11 differentially expressed miRNAs of the T1D male cohort.

**
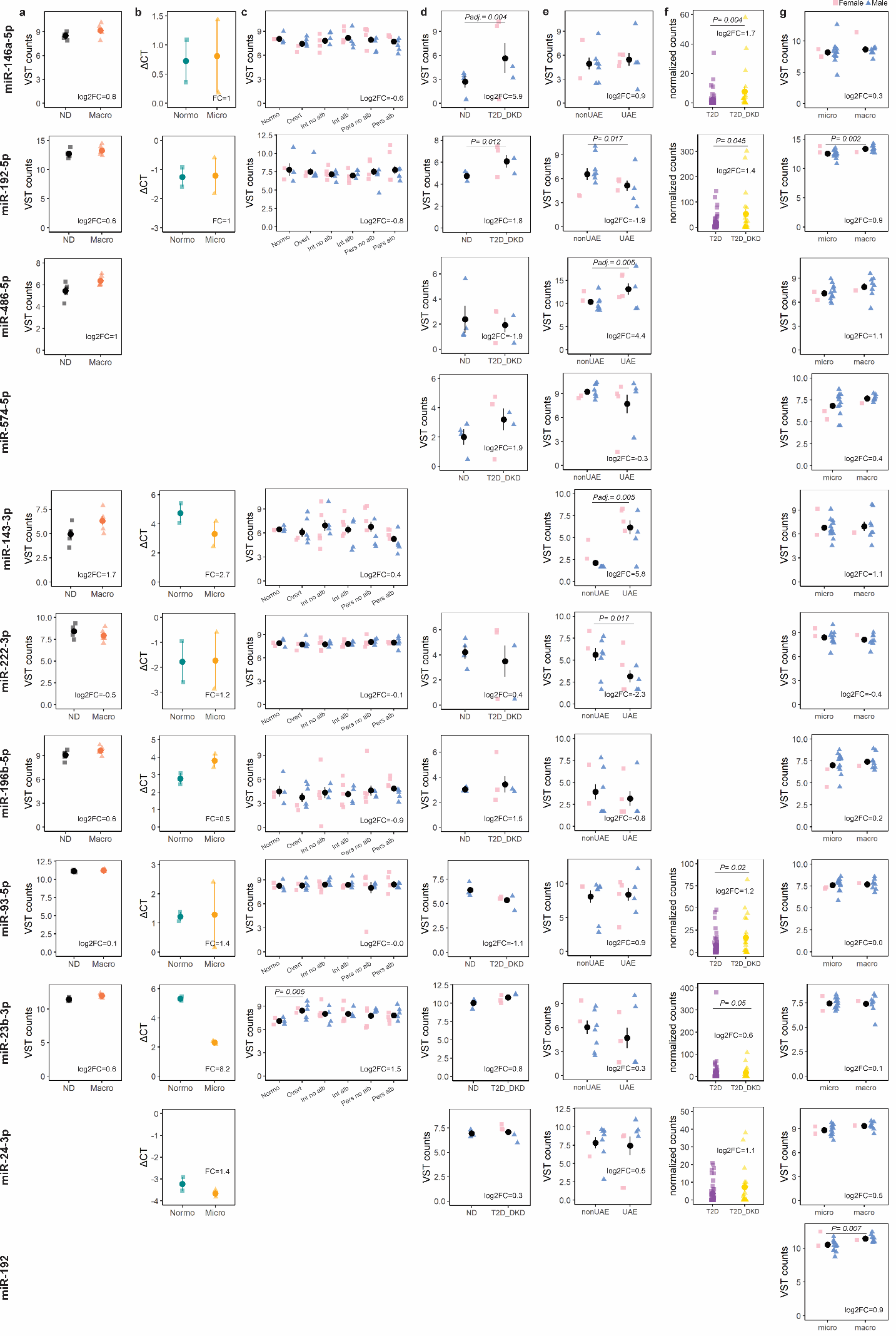
**

**Supplementary Figure S5:** Replication of findings using miRNA profiling datasets of uEV in DKD. (a) T1D from Barreiro et al, 2020 (dataset derived from uEV isolated by ultracentrifugation). (b) T1D from Barutta et al. 2015. (c) T1D from Ghai et al, 2017. Note: the FC informed here corresponds to Overt vs Normo group comparison, we did not study the other groups. (d) T2D from Park et al, 2020 and 2022. (e) T2D subpopulation from Perez-Hernandez et al, 2021. (f) Extended T2D cohort from Ali et al. 2024, where all sequenced samples have been presented. Note that the log2-foldchange and p-values were calculated after removing non-zero values and clear outliers in a prefiltering step by the authors. (g) this study, T2D cohort. A higher or lower expression in the case vs control group was considered if the log2FC in sequencing was ≥0.6 or ≤-0.6, or the FC in qPCR was ≥2 or ≤-0.5, respectively. Diabetic kidney disease (DKD), fold change (FC) Intermitent albuminuria (Int alb), intermittent non-albuminuria (Int non alb), macroalbuminuria (Macro), microalbuminuria (Micro), non-diabetic (ND), normoalbuminuria (Normo), p adjusted value (Padj.), persistent albuminuria (Pers alb), persistent non-albuminuria (Pers non alb), type 2 diabetes (T2D), Type 2 diabetes and diabetic kidney disease (T2D-DKD), urinary extracellular vesicles (uEV), urine albumin excretion (UAE), variance stabilizing transformation (VST). Details on samples included in the different datasets is provided in Table 4.

**
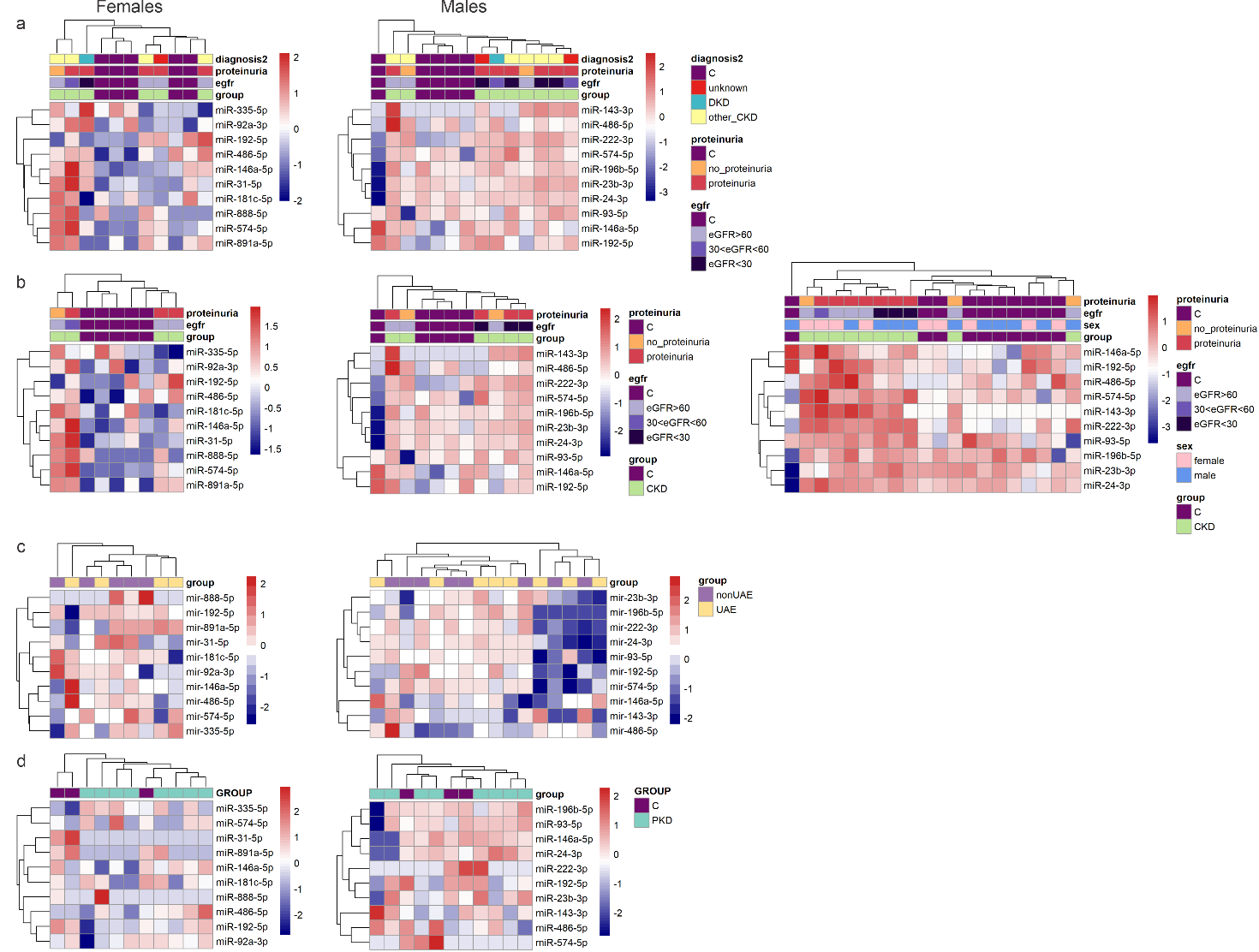
Supplementary Figure S6:** Heatmap with hierarchical clustering of control published uEV datasets based on the VST normalized read counts of the DE miRNAs in the T1D cohorts with sex stratification. Heatmaps depict the clustering of the studies presented on Figure 7 separated by sex (for the studies where that information was available): clustering of females has been presented based on DE miRNAs from the female T1D replication cohort and clustering of males and combined cohorts has been presented for the DE miRNAs from the T1D male discovery study. a and b) CKD, Khurana et al, 2017 in b, clustering performed without individuals with DKD or unknow diagnosis. c) Hypertension, Perez-Hernandez 2021, d) PKD, Magayr et al. 2020. Chronic kidney disease (CKD), control (C), diabetic kidney disease (DKD), estimated glomerular filtration rate(eGFR), no urinary albumin excretion (non-UAE), polycystic kidney disease (PKD), with urinary albumin excretion (UAE).


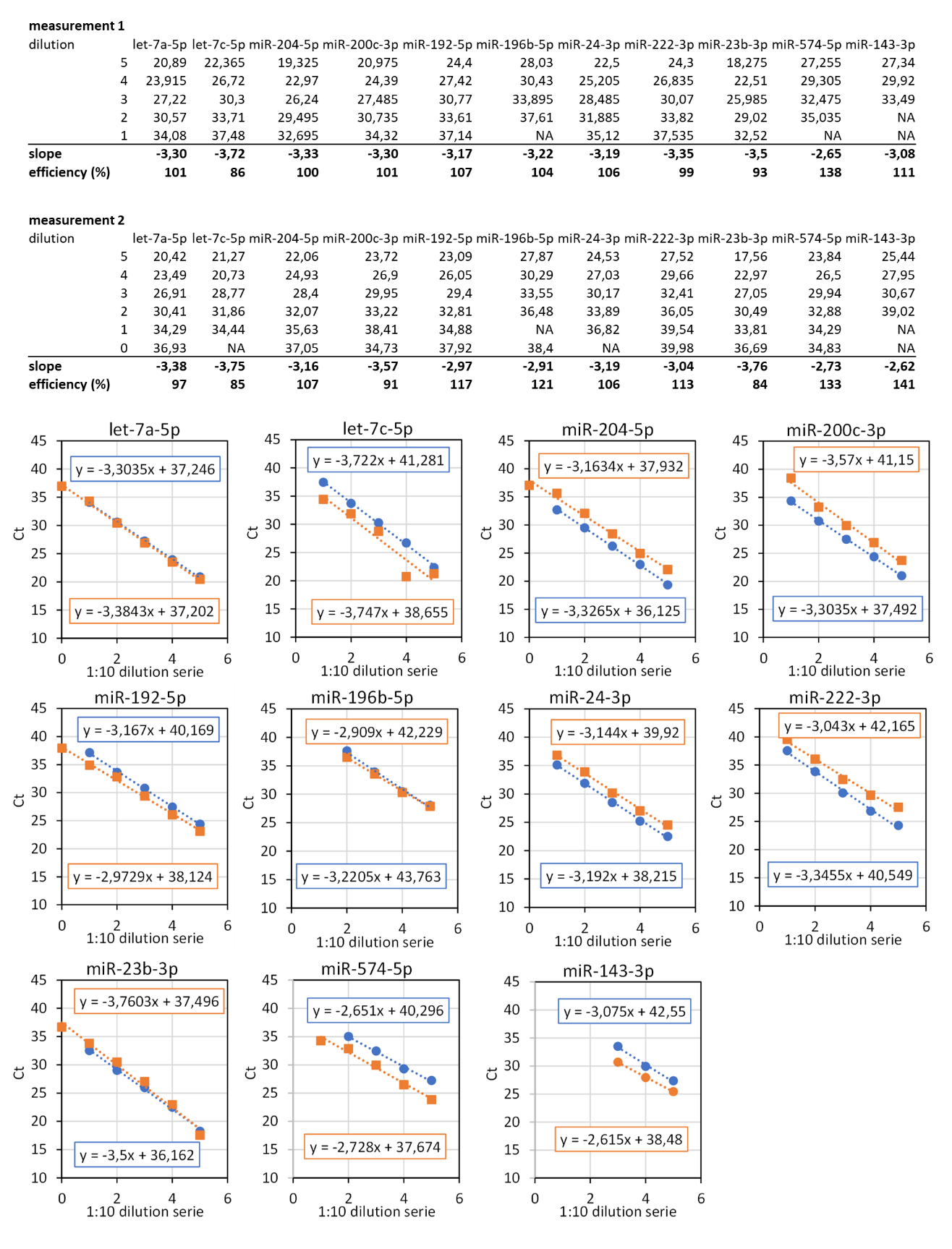
**Supplementary Figure S7:** qPCR primer efficiency. The efficiency of the miRNA advanced assays was tested with two dilution series (in blue and orange). The slope value was calculated using excel and fitted trendline. Clear outlier values from the highest dilutions were left out from the plot. The efficiency was calculated from the slope using a formula: E = -1+10(-1/slope)*100. Cycle threshold (ct).
